# Development of High-Throughput Sample Preparation Procedures for the Quantitative Determination of Aflatoxins in Biological Matrices of Chickens and Cattle Using UHPLC-MS/MS

**DOI:** 10.3390/toxins15010037

**Published:** 2023-01-03

**Authors:** Siegrid De Baere, Phillis E. Ochieng, David C. Kemboi, Marie-Louise Scippo, Sheila Okoth, Johanna F. Lindahl, James K. Gathumbi, Gunther Antonissen, Siska Croubels

**Affiliations:** 1 Laboratory of Pharmacology and Toxicology, Department of Pathobiology, Pharmacology and Zoological Medicine, Faculty of Veterinary Medicine, Ghent University, Salisburylaan 133, 9820 Merelbeke, Belgium; 2 Laboratory of Food Analysis, FARAH—Veterinary Public Health, University of Liège, Avenue de Cureghem 10, 4000 Liège, Belgium; 3 Department of Veterinary Pathology, Microbiology, and Parasitology, Faculty of Veterinary Medicine, University of Nairobi, P.O. Box 29053, Nairobi 00100, Kenya; 4 Department of Animal Sciences, Chuka University, P.O. Box 109-60400, Chuka 00625, Kenya; 5 Department of Biology, Faculty of Science and Technology, University of Nairobi, P.O. Box 30197, Nairobi 00100, Kenya; 6 International Livestock Research Institute (ILRI), P.O. Box 30709, Nairobi 00100, Kenya; 7 Department of Medical Biochemistry and Microbiology, Uppsala University, SE-751 05 Uppsala, Sweden; 8 Department of Clinical Sciences, Swedish University of Agricultural Sciences, SE-750 07 Uppsala, Sweden; 9 Chair Poultry Health Sciences, Faculty of Veterinary Medicine, Ghent University, Salisburylaan 133, 9820 Merelbeke, Belgium

**Keywords:** aflatoxins, chicken, cattle, UHPLC-MS/MS, biological matrices, residues, food safety, toxicokinetics

## Abstract

Aflatoxins (AFs) frequently contaminate food and animal feeds, especially in (sub) tropical countries. If animals consume contaminated feeds, AFs (mainly aflatoxin B1 (AFB1), B2 (AFB2), G1 (AFG1), G2 (AFG2) and their major metabolites aflatoxin M1 (AFM1) and M2 (AFM2)) can be transferred to edible tissues and products, such as eggs, liver and muscle tissue and milk, which ultimately can reach the human food chain. Currently, the European Union has established a maximum level for AFM1 in milk (0.05 µg kg^−1^). Dietary adsorbents, such as bentonite clay, have been used to reduce AFs exposure in animal husbandry and carry over to edible tissues and products. To investigate the efficacy of adding bentonite clay to animal diets in reducing the concentration of AFB1, AFB2, AFG1, AFG2, and the metabolites AFM1 and AFM2 in animal-derived foods (chicken muscle and liver, eggs, and cattle milk), chicken and cattle plasma and cattle ruminal fluid, a sensitive and selective ultra-high performance liquid chromatography-tandem mass spectrometry (UHPLC-MS/MS) method has been developed. High-throughput sample preparation procedures were optimized, allowing the analysis of 96 samples per analytical batch and consisted of a liquid extraction using 1% formic acid in acetonitrile, followed by a further clean-up using QuEChERS (muscle tissue), QuEChERS in combination with Oasis^®^ Ostro (liver tissue), Oasis^®^ Ostro (egg, plasma), and Oasis^®^ PRiME HLB (milk, ruminal fluid). The different procedures were validated in accordance with European guidelines. As a proof-of-concept, the final methods were used to successfully determine AFs concentrations in chicken and cattle samples collected during feeding trials for efficacy and safety evaluation of mycotoxin detoxifiers to protect against AFs as well as their carry-over to animal products.

## 1. Introduction

Aflatoxins (AFs) are secondary metabolites produced by *Aspergillus* fungi, which frequently contaminate agricultural crops, especially in developing countries with warm climates, poor food quality, safety control, production technologies, and bad crop storage conditions [1,2,3]. Given their hepatotoxic, immunosuppressive, and potent carcinogenic properties, AFs are among the most important mycotoxins. There are four main types of naturally occurring aflatoxins: aflatoxin B1 (AFB1), B2 (AFB2), G1 (AFG1), and G2 (AFG2), which are considered toxic components and classified as group 1 carcinogens by the International Agency for Research on Cancer (IARC) [4,5,6]. In mammals and poultry, AFB1 and AFB2 can be bio-transformed into aflatoxin M1 (AFM1, group 1) [4,7] and aflatoxin M2 (AFM2). The toxicity of these aflatoxins can be ranked as AFB1 > AFG1 > AFB2 > AFG2, the latter two being less carcinogenic and less mutagenic than AFB1 [2,8]. 

Mycotoxins can occur in food, either by direct contamination (plant materials or products thereof) or by carry-over of these components and their metabolites into tissues, milk, and eggs of animals that consumed contaminated feed [1,9,10,11,12]. The impact of mycotoxins on human and animal health depends on the type of mycotoxin, ingested amounts, exposure duration, synergisms in toxicity if different mycotoxins are ingested simultaneously, animal species, gender, age, as well as the status of the immune system and general health [2,10,12]. Mycotoxins present in poultry feed can have negative effects on poultry health and, as a consequence, impact productivity. Moreover, human health can be at risk by secondary exposure to AFs, i.e., through the consumption of food products (eggs, liver, and meat) from chickens fed AFs-contaminated feed [12]. In ruminants, a part of the ingested AFB1 is degraded in the rumen, whereas the remaining fraction is absorbed in the gut and metabolized in the liver to aflatoxin M1 (AFM1), which is subsequently transferred to the milk [3]. Studies have indicated that the biotransformation rate can vary between animals and other factors, such as nutritional and physiological status, diet, ingestion and digestion rate, animal health, liver biotransformation capacity, and level of milk production [3,9]. In healthy ruminants, mycotoxins can be degraded by the rumen microbiota, which reduces the risk of milk contamination. However, animal diseases, changes in the diet, or high mycotoxin contamination levels can alter the rumen barrier. Moreover, the ruminal fluid, which is seen as the first defense mechanism against mycotoxins such as zearalenone, ochratoxin A, T-2 toxin, and diacetoxyscirpenol, is not fully effective for AFB1, and patulin [9,13]. The ruminal conditions and the rumen microbiome metabolism can be altered by certain cow diseases, such as rumen acidosis [14] and/or high contamination of mycotoxins in feed, which favor the carry-over of mycotoxins in milk, especially AFs [15].

Due to its thermal stability, the concentration of AFM1 is not affected by manufacturing processes [16]. Therefore, the most effective method to control the AFM1 concentration in milk is applying good agricultural practices (GAP) to reduce AFB1 contamination of raw materials and cattle feed [9]. Post-harvest intervention strategies can also be applied to reduce animal and human AFs exposure, such as dehulling, fermentation, nixtamalization, or the usage of mycotoxin modifiers and/or binders [16,17]. In addition, regulations have been developed for AFB1 and total AFs (sum of AFB1, AFB2, AFG1, and AFG2) in food and feeds by several countries [3,7,8,12,16]. The highest acceptable level of AFM1 in milk set by the European Union (EU) and the United States Food and Drug Administration (USFDA), respectively, ranges between 0.05 µg kg^−1^ and 0.5 µg kg^−1^ [18,19], while the highest allowed level is 20 µg kg^−1^ for total AFs in foods for human consumption [20]. In addition, many countries have established maximum permissible levels of AFM1 in milk (range: 0–10 µg kg^−1^) and dairy products (range: 0–10 µg kg^−1^) to reduce risks [3,9]. In contrast, there is no specific legislation for the occurrence of AFs in chicken tissues and eggs [1,12]. 

Commodities and feed are usually analyzed to diagnose mycotoxicosis in animals and assess animal exposure to mycotoxins. However, there are several disadvantages of estimating mycotoxin intake in this way: inhomogeneous distribution of mycotoxins among a feed lot, failure to measure the exposure at the individual level, and occurrence of modified or conjugated forms. These create possible mismatches between feed contamination levels and exposure to animals [21,22]. The relevant mycotoxin exposure can be evaluated by the analysis of so-called biomarkers of exposure in different biological matrices of chickens (plasma, tissues, eggs) and cattle (plasma, ruminal fluid, milk). These biomarkers are often the parent mycotoxin itself, in vivo formed phase I or phase II metabolites or adducts with macromolecules, such as nucleic acids and proteins [2,22]. The selection of a biomarker to be analyzed in a certain biological matrix is crucial.

The purpose of the analysis of AFs in biological samples can be different. Analysis of AFs in plasma can be performed as a part of toxicokinetic, ADME (absorption, distribution, metabolization, excretion), and bioavailability studies, and to assess exposure. Analyzing AFs in plasma can also be used to evaluate the in vivo efficacy of mycotoxin detoxifiers according to the guidelines of the European Food Safety Authority (EFSA) [23]. Analysis of AFs in animal tissues, milk, and eggs can be performed to investigate the tissue distribution, bioaccumulation, persistence, and carry-over of AFs in different animal species [10].

To meet official regulations and food safety concerns, analytical methods are needed that are accurate and sensitive enough to allow the quantitative determination of AFs in food, feed, and biological matrices [8]. The analysis of mycotoxins in biological samples is a challenge because these are generally complex matrices consisting of lipids, proteins, and carbohydrates. Widely applied methods for the determination of AFs are based on enzyme-linked immunosorbent assays (ELISA), and high-performance liquid chromatography (HPLC) coupled to ultraviolet (UV) or fluorescence detection (FLD) [7,8,24,25,26]. However, HPLC-UV/FLD methods generally need tedious sample preparation and pre- or post-column derivatization steps, whereas ELISA methods may show potential cross-reactivity with metabolites of the analytes of interest or matrix components [7,11,25]. In recent decades, (ultra)high-pressure liquid chromatography ((U)HPLC) coupled with mass spectrometry (MS) has become the standard method for the analysis of mycotoxins in food, feed, and other matrices due to its superior specificity and sensitivity [5,6,8,27,28,29,30]. Either triple quadrupole MS instruments enabling tandem mass spectrometry (MS/MS), which provide high sensitivity and specificity [1,28], or high-resolution mass spectrometry [31,32] are used.

When using these methods, the sample preparation procedure is critical, particularly for complex matrices, such as plasma, organs and tissues, milk, and eggs [5]. Sample matrix components that co-elute with the analytes of interest may have a serious impact on the ionization process, causing signal suppression/enhancement (SSE) [27,29]. Pretreatment generally consists of a liquid extraction step, followed by clean-up steps to reduce or remove co-extracted matrix components, such as proteins, sugars, and lipids [1,8,10,15,25,28,33,34]. 

AFs isolation from plasma is often based on generic and simple liquid extraction (LE) methods using acetonitrile (ACN), methanol, or a mixture of these solvents with water, alone or in combination with an organic acid (formic acid (FA), acetic acid (AA)) [5]. Another generic extraction procedure that is applied, especially for multi-mycotoxin procedures, is the QuEChERS protocol (acronym of Quick, Easy, Cheap, Effective, Rugged, and Safe). The method consists of the extraction with acidified ACN, followed by the liquid-liquid partition of analytes after the addition of salts [1,5,35]. More complex matrices, such as milk, solid biological matrices (organs and tissues), or eggs, are generally subjected to liquid-liquid extraction (LLE) or liquid-solid extraction (LSE) techniques with several solvents (ACN, ethyl acetate) [5,8,10,36,37]. In some cases, a further clean-up is needed to remove co-extracted matrix components, which can consist of conventional solid-phase extraction (SPE) or expensive but highly selective immunoaffinity columns (IAC) [1,8,10,25]. Some authors also include a hexane defatting step to remove fat components which are present in the matrix and could interfere with the detection process [10].

The goal of the current study was threefold: (1) to develop high-throughput sample preparation procedures to allow the processing of ≥96 samples per day. Moreover, a UHPLC-MS/MS method was optimized to reach maximal sensitivity and selectivity, which was needed for the analysis of generally low AFs concentrations (lower ng mL^−1^ or µg kg^−1^ level) in various biological samples of chickens (plasma, tissues, eggs) and cattle (plasma, rumen fluid, and milk); (2) secondly, the optimized methods were in-house validated to evaluate their suitability for routine application; (3) finally, the applicability of the methods was tested by the analysis of incurred samples that were taken as a part of in vivo efficacy studies with an AFB1-specific mycotoxin detoxifying agent in cattle, broiler chickens and layers, receiving either a control diet or an AFs contaminated diet. In such a way, it could be demonstrated that the final methods can be applied in four areas of expertise: assessing animal exposure to AFs; assessing the efficacy of candidate mycotoxin detoxifiers; monitoring food safety; and evaluating the toxicokinetics, ADME, and bioavailability characteristics of individual AFs.

## 2. Results and Discussion

### 2.1. Sample Preparation

Sample preparation was a crucial step in method development for the analysis of AFs in different biological matrices of animal origin. High extraction recovery and limited matrix effects on the UHPLC-MS/MS instrument were desirable since AFs are expected to be present in low concentrations (~pg mL(g)^−1^ to ng mL(g)^−1^). Since the final aim was to analyze a high number of samples per day (n ≥ 96, including incurred samples, calibrator, blank, and quality control samples), the sample preparation procedure had to be straightforward and preferably cost-effective. Therefore, several types of high-throughput sample preparation techniques were investigated during method optimization, depending on the biological matrix, i.e., protein precipitation (PPT), LE + QuEChERS, LE + SPE, LE + QuEChERS + SPE.

#### 2.1.1. Plasma

The current sample preparation method used for AFs in chicken plasma was based on that of Lauwers et al. for the analysis of 24 mycotoxins [22]. The generic sample preparation procedure used an Ostro^TM^ 96-well plate to combine PPT with 1% FA in ACN with phospholipids (PL) removal. PL are reported to be present in significantly higher concentrations in chicken plasma compared to other species (pigs, cows, horses, and ostriches) [38]. Moreover, Chambers et al. [39] indicated that matrix components and PL, in particular, can be an important source of imprecision in quantitative UHPLC-MS/MS analysis. The same procedure was applied for the analysis of cattle plasma due to the ease of use of the overall Ostro^TM^ 96-well plate protocol, which allows the simultaneous extraction of 96 plasma samples within 2 h. Methods reported by other authors for the analysis of AFs in serum or plasma are based on PPT using ACN alone [21] or in combination with an organic acid (1% AA [5]; 0.1–1.0% FA [22,24]). Some authors combine PPT with some type of SPE [22] or IAC [40], while others report LLE using ethyl acetate [33]. Some authors combined a LE step with SPE clean-up [27].

#### 2.1.2. Milk and Ruminal Fluid

Milk is a complex matrix that contains a.o. proteins, fats, and sugars, which could have a negative impact on extraction recovery and/or produce matrix effects. Some authors perform a simple extraction with ACN/water (50/50, v/v), followed by centrifugation and filtration before LC-MS/MS analysis [29]. However, most procedures include a clean-up step after the initial LE. Direct IAC was used for AFM1 extraction from cow milk [41]. In human breast milk, sample preparation consisted of LLE using combinations of solvents such as chloroform, ACN [7,34,42], or ethyl acetate [34]. The latter authors observed a better recovery using LLE compared to SPE, with the highest recovery provided by a mixture of acidified ACN and ethyl acetate [34]. Flores-Flores et al. obtained the best results with ACN, to which 2% FA was added [15]. The authors mentioned that FA was needed in the extraction step to enhance mycotoxin migration to the organic phase. QuEChERS techniques were also applied [13,15,36,43] to enhance the phase separation between the aqueous milk matrix and the organic phase (ACN) and to increase the removal of matrix components. Hexane and chloroform are sometimes used to remove milk fat. 

Different extraction procedures were tested for AFB1 and AFM1 during preliminary experiments (results not shown): protein precipitation + liquid extraction (PPT + LE), LE in combination with QuEChERS (LE + QuEChERS), LE in combination with Oasis^®^ PRiME HLB SPE clean-up (LE + PRiME) and LE in combination with QuEChERS and Oasis^®^ PRiME HLB SPE clean-up (LE + QuEChERS + PRiME). The LE + PRiME procedure was selected as the final procedure because of the ease of use of this pass-through SPE cleanup. The Oasis PRiME (abbreviation of process, robustness, improvements, matrix effects, ease of use) HLB cartridges were first introduced by Waters for multiclass, multi-residue LC-MS/MS screening of veterinary drugs in meat (water 70%, protein 15–25%, fat 5–25%, PL 1–3%). This procedure is highly effective in the removal of both fat and PL from meat extracts. It has the advantage that no cartridge conditioning is required, and that sample clean-up using the Oasis PRiME HLB cartridge is based on the pass-through principle, allowing the analysis of a large number of samples in one analytical batch (n ≥ 96). The final sample preparation procedure for milk was also tested on ruminal fluid. Acceptable results for extraction recovery (R_E_, %, range: 24.7–47.8% for milk; 61.4–75.1% for ruminal fluid) and matrix effect (M_E_, %, range: 53.3–80.8% for milk; 21.8–37.2% for ruminal fluid) were obtained for the tested AFs in both matrices (see Table S10).

#### 2.1.3. Edible Animal Tissues

Edible animal tissues are complex matrices containing fats, proteins, carbohydrates, and inorganic salts [5]. The first sample preparation step consisted of a LE using 3 mL of 1% FA in ACN, which is in accordance with methods reported by other authors [24,44]. However, for muscle tissue, it was important to add 2 mL of water to the tissue matrix since direct extraction with 1% FA in ACN resulted in serious agglomeration of the sample [44]. Zhao et al. observed that ACN was the most suitable solvent for extraction of multiple mycotoxins from animal-derived food and milk because extraction recoveries were higher and co-extraction of interferences lower, compared to MeOH or acetone [44]. The same findings were observed by Chen et al. [11]. Moreover, the acidification of ACN with FA or AA improved the extraction recovery of some mycotoxins, such as fumonisins and mycophenolic acid, which makes it a suitable solvent for the extraction of mycotoxins from other classes than AFs (= potential future application of the presented method). 

Liquid extraction in combination with QuEChERS is preferred by many authors as the first step in sample clean-up for mycotoxins in biological matrices [1,5,35,44,45]. The use of QuEChERS salts improves the transfer of AFs to the organic phase and allows for a better phase separation between the aqueous tissue matrix and the organic extraction solvent. 

*Muscle.* During preliminary experiments, it was observed that further sample clean-up of the muscle extract using a PRiME HLB SPE column (60 mg/3 cc) did not result in a substantial improvement in apparent recovery. Therefore, it was decided to use a combination of LE and QuEChERS for the extraction of AFs from muscle tissue. The sensitivity of the method could not be improved by including a sample concentration step (i.e., evaporating the sample extract, followed by redissolution of the dry residue in a limited volume of solvent) due to an increase in signal suppression on the UHPLC-MS/MS instrument (results not shown). After transferring 500 µL of the final sample extract to an autosampler vial, 200 µL of ULC/MS water was added, followed by the direct injection of a 5-µL aliquot onto the UHPLC-MS/MS instrument. In this way, a time-consuming evaporation step could be avoided, reducing the total sample preparation time by at least 1–1.5 h.

*Liver.* Most mycotoxins target the liver, and in addition, the biotransformation of these mycotoxins through metabolic processes generally occurs in the liver of animals, and thus higher levels have been detected in liver samples compared to other edible animal tissues [46,47]. The liver is also a complex matrix like the muscle and thus consists of fatty compounds, proteins, and mineral substances. Therefore, sample pre-treatment is critical so as to improve method sensitivity. The method developed in the current study involved an extraction step using 3 mL of 1% FA in ACN, followed by a first clean-up step using QuEChERS. These QuEChERS methods have been used successfully to extract mycotoxins in liver samples from chickens [5] and pigs [44]. A further clean-up step with an Oasis Ostro^TM^ 96-well plate was employed in the current method to remove the PL co-extracted with the analytes of interest. In the methods reported in other studies [5,44], SPE in combination with a hexane defatting step was used to remove the fatty compounds. 

*Eggs.* Similar to the liver and tissues, eggs are complex food matrices consisting of fatty compounds, proteins, cholesterol, vitamins, and mineral substances that may be co-extracted with AFs during the extraction step. Moreover, mycotoxins have been reported to occur in eggs at trace levels [35,37,48,49,50,51] and are generally considered to be mainly present in the yolk part of the eggs if they are hydrophobic [37,51). However, in this study, the whole egg was analyzed as it is often consumed as such. Extraction was achieved using 1% FA in ACN, and to enhance sensitivity, the supernatant was evaporated to dryness before being reconstituted. The use of acidified ACN has been reported as an effective way of extracting mycotoxins from eggs [35]. The method developed in this study was eco-friendlier as it used low extraction solvent (3 mL of 1% FA in ACN) as compared to methods reported by Frenich et al. [1] (10 mL of 1% AA in MeOH/water solution (80/20, v/v)) and Zhu et al. [35] (5 mL of 1% FA in ACN). An additional clean-up step using a commercial Oasis Ostro^TM^ 96-well plate was used in the present study to minimize interferences by co-extracted matrix components. This step enabled the method to achieve lower LOQs and LODs compared to methods reported by Frenich et al. [1] and Zhu et al. [35]. QuEChERS extraction was not employed in this method but has been successfully employed in methods developed by other authors [1,35,49,51] for multi-mycotoxin analysis of eggs. One disadvantage noted with the use of QuEChERS is the aggregation of the salts [1,51], although better recovery efficiencies were achieved in the latter studies compared to the present study. 

By using internal standardization for all procedures, analyte losses during sample preparation and matrix effects during UHPLC-MS/MS analysis could be compensated for. Three isotope-labeled ISs (^13^C_17_-AFB1, ^13^C_17_-AFM1, ^13^C_17_-AFG1) were chosen because their physico-chemical and structural properties are very similar to AFB1, AFM1, and AFG1, respectively. No isotope-labeled ISs were used for AFB2, AFG2, and AFM2 to reduce the cost of the analysis. These analytes were quantified using ^13^C_17_-AFB1 (AFB2 and AFG2) and ^13^C_17_-AFM1 (AFM2) as internal standards. Although this was theoretically not optimal, method validation results showed that the reliable determination of these components was not impaired. Some authors added the IS after the extraction procedure but prior to LC-MS/MS analysis, which compensates only for matrix effects on the LC-MS/MS instrument [36]. No ISs were used by Flores-Flores et al., although it was observed that mycotoxins had different behaviors in terms of their recoveries and matrix effects depending on the type of milk matrix (e.g., semi-skimmed evaporated versus whole UHT cow milk) [13].

### 2.2. Liquid Chromatography

This study aimed to develop a UHPLC-MS/MS method for the analysis of the major AFs within an acceptable run-time (10 min), to allow the analysis of ≥96 samples in a 24-h period. It was important to separate the isobaric compounds AFG1/AFM1 and AFG2/AFM2 (see Table 1). In addition, the mobile phases and modifiers were chosen in order to obtain maximal sensitivity. 

Chromatography was performed using a UPLC HSS T3 column (100 × 2.1 mm, dp: 1.8 µm) since this was also used with success by Lauwers et al. and Braun et al. for the chromatographic separation of multiple mycotoxins [22,36]. Based on the literature [52] and our findings during preliminary experiments, a better sensitivity was obtained for all AFs using a combination of water/MeOH, compared to water/ACN as mobile phases (results not shown). Secondly, the effect of different organic modifiers on signal intensity and separation of isobaric compounds was evaluated. Four aqueous mobile phases (MF) were tested, i.e., water without any modifiers (MF-1), 5 mM ammonia formate (NH_4_FA) + 0.1% FA in water (MF-2) [52], 10 mM NH_4_FA + 0.3% FA in water (MF-3) [22] and 5 mM ammonia acetate (NH_4_AA) + 0.1% AA in water (MF-4) [36], in combination with MeOH. As can be seen from Figure S1, the best signal intensities (based on peak area) were obtained with water without the addition of any modifier. The addition of a combination of NH_4_FA and FA resulted in higher peak areas compared to NH_4_AA and AA. It was aimed to determine the AFs in animal biological matrices at levels that are as low as possible and therefore it was finally decided to add no organic modifiers to the aqueous or organic mobile phase.

**Figure 1:**
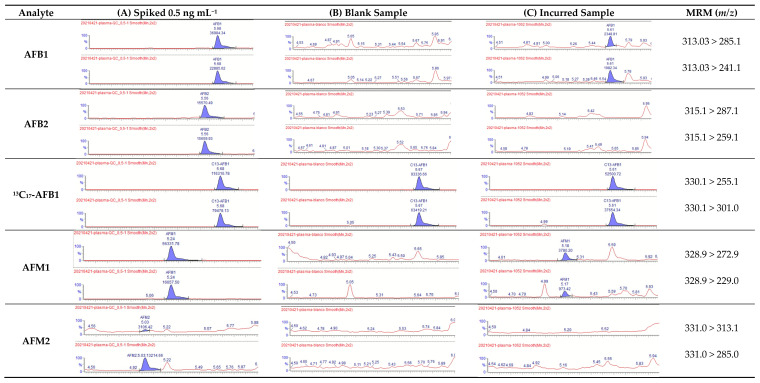

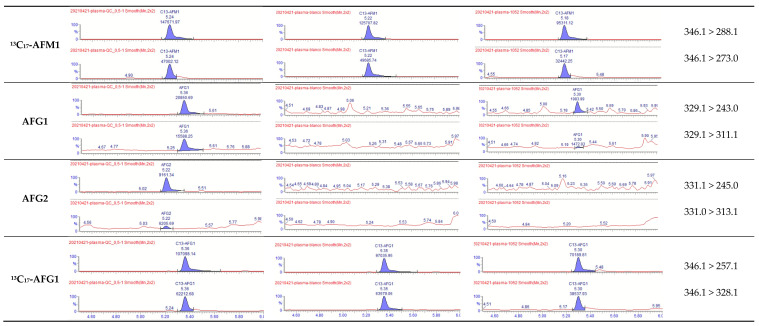
UHPLC-MS/MS chromatograms of (**A**) a blank chicken plasma sample spiked with aflatoxins at a concentration of 0.5 ng mL^−1^; (**B**) a blank chicken plasma sample spiked with the ISs; (**C**) an incurred chicken plasma sample (AFB1: 0.078 ng mL^−1^; AFM1: 0.069 ng mL^−1^; AFG1: 0.086 ng mL^−1^).

Gradient elution was performed. The total run-time was 10 min, which was acceptable for application in the research field of toxicokinetic and detoxifiers efficacy studies and residue analysis. This was comparable to other methods reported for the analysis of AFs in biological matrices (5 min (only AFB1) [5]; 7 min [27]; 8 min [21], AFB1-specific method; 10 to 15 min for AFs in milk and animal feed [28]). As can be seen from Figure 1A, a good separation was obtained between the isobaric compounds AFM1 and AFG1 ([M + H]^+^ *m/z* 329.1) and AFM2 and AFG2 ([M + H]^+^ *m/z* 331.1). In a blank chicken plasma sample, no peaks were present at the elution zone of the analytes of interest (Figure 1B) and in other biological matrices (results not shown), indicating the good specificity of the presented UHPLC-MS/MS method.

### 2.3. Mass Spectrometry

Fluorescence detectors are often used for the analysis of AFs in different matrices due to the native fluorescence properties of these components and because of the high sensitivity and specificity of these types of detectors. However, AFB2 and AFG2 are naturally strongly fluorescent molecules, while AFB1 and AFG1 need pre- or post-column derivatization to improve fluorescent properties and, as a consequence, method sensitivity [12,13].

The issues related to pre- or post-column derivatization can be overcome by the use of UHPLC-triple quadrupole mass spectrometric (MS/MS) instruments, resulting in enhanced selectivity and high sensitivity. The AFs were detected in the multiple reaction monitoring (MRM) mode, with the electrospray ionization (ESI) source operating in the positive mode. For each precursor ion, the two most abundant product ions were selected for quantification and identification purposes, respectively (Table 1). [M + H]^+^ ions were selected as precursor ions since it was observed by Chen et al. that fragments of [M + NH_4_]^+^ and [M + Na]^+^ precursor ions were not stable [11]. The two most abundant product ions for AFB1, AFB2, AFG1, AFG2, AFM1, and AFM2 were selected as quantifiers and qualifier ions in the final method. Selected product ions are mentioned in Table 1 and were the same as reported in the literature [6,11,21,27,29,44].

### 2.4. Method Validation

The results of the method validation experiments for chicken plasma are shown in Table 2 and Table 3. The validation results for AFs in the other biological matrices (cattle plasma, milk, and ruminal fluid; chicken liver, muscle, and eggs) are shown in Supplementary Tables S2–S17. 

#### 2.4.1. Linearity

Matrix-matched calibrator samples were used to construct calibration curves in order to compensate for matrix effects. Calibration curves were linear and covered a concentration range of LOQ to 200 ng mL^−1^ (chicken plasma) and LOQ to 10 ng mL(g)^−1^ (other matrices) (see Table 2; Tables S2 and S3). A weighting factor of 1/x^2^ was used for all calibration curves. The correlation coefficients (r) and goodness-of-fit coefficients (g) fulfilled the acceptance criteria and were ≥0.99 and ≤20%, respectively. Other authors reported calibration curves in the same range for human plasma [33,53], pig plasma [6,21,27], and pig and chicken plasma [22]. Calibration curves in milk ranged between 0.05–30 ng mL^−1^ in human breast milk [36] and 0.02–20 ng mL^−1^ for AFM1 in cow milk [7]. For eggs, calibration ranges between 1 and 200 µg kg^−1^ [1] and 0.2 and 100 µg kg^−1^ [35] were reported for different aflatoxins, whereas for swine liver between 0.05–50 µg kg^−1^ for AFB1 and AFB2, 0.1–100 µg kg^−1^ for AFG1, AFG2 and AFM1 [5]. These results demonstrated the suitability of the current method for screening for AFs in different matrices, especially in chronic exposure to these mycotoxins.

#### 2.4.2. Accuracy and Precision

The within-run accuracy and precision were tested at the LOQ level (0.025–0.50 ng mL(g)^−1^) and at two or three different concentration levels (i.e., low, 0.5 ng mL(g)^−1^, medium, 5 ng mL(g)^−1^ and high, 50 ng mL(g)^−1^; the latter concentration level was only evaluated in chicken plasma). The acceptance criteria were fulfilled for all compounds at the specified levels. The between-run precision and accuracy were evaluated at the same concentration levels, and the results also fell within the predefined ranges (see Table 3, chicken plasma; Table S4, cattle plasma; Table S6, cattle milk; Table S8, cattle ruminal fluid; Table S11, chicken liver; Table S13, chicken muscle; Table S15, chicken eggs).

#### 2.4.3. Limit of Quantification (LOQ) and Limit of Detection (LOD)

LOQ values ranged between 0.025–0.50 ng mL^−1^ in chicken and cattle plasma and cattle milk; 0.10–0.50 ng mL^−1^ in cattle ruminal fluid; 0.05–0.25 µg kg^−1^ in chicken muscle; 0.05–0.50 µg kg^−1^ in chicken liver; 0.025–0.50 µg kg^−1^ in chicken eggs, depending on the aflatoxin. The calculated LOD values ranged between 0.002 and 0.060 ng mL^−1^ (chicken and cattle plasma); 0.002 and 0.038 ng mL^−1^ (cattle milk); 0.012 and 0.132 ng mL^−1^ (cattle ruminal fluid); 0.006 and 0.040 µg kg^−1^ (chicken tissues); 0.002 and 0.097 µg kg^−1^ (chicken eggs). These LOQ values fell in the same range or even lower than those mentioned by other authors: 0.01–2.0 ng mL^−1^ in rat plasma [24,27]; 0.1–0.8 ng mL^−1^ for AFs in human plasma or serum [5,6,33,53]; 1 ng mL^−1^ for AFB1 and AFM1 in pig and chicken plasma [21,22]; 2 µg kg^−1^ for AFB1, AFB2, AFG1 and AFG2 in chicken liver [54]; 0.12–0.25 µg kg^−1^ in swine liver [5]; 0.05–8 µg kg^−1^ in rat tissues [24,27]. The lower or same range of LOQ values was also achieved in this study for different AFs in eggs as compared to those achieved by other authors (ranging between 0.2 and 2.0 µg kg^−1^) [1,35,37,54]. Moreover, LOD values for AFs in eggs achieved in the current study were lower than those specified in the literature [1,35,37,54], implying that the current method could be used to analyze AFs in eggs at trace levels and applied to food control schemes. Lower LOD values were achieved for different AFs in the liver in the present study compared to the method developed by Wang et al. (LOD: 1 µg kg^−1^), implying the suitability of the current method for analysis of AFs in food at trace levels [54].

The EU legislation has set a maximum allowed level of 0.05 µg kg^−1^ for AFM1 in milk. Therefore, LOQ and LOD values for AFs in milk should be as low as possible. The LOQ values for AFs in milk obtained with the presented method ranged between 0.025 and 0.05 µg kg^−1^, which is sufficiently low to detect these contaminants at the maximum allowed level by the EU (except for AFM2). LOQ values for AFs reported by other authors ranged between 0.08–0.16 ng mL^−1^ in human breast milk [36]; 0.02–0.50 ng mL^−1^ in cow milk [7,13,28,44].

LOQ values in pork meat ranged between 0.1–0.2 µg kg^−1^ [44] and between 0.25–1 µg kg^−1^ in various animal-derived food matrices [11].

The obtained LOQ and LOD values in all matrices were sufficiently low, and hence it was possible to quantify the analytes of interest accurately in samples that were taken from laying hens, broiler chickens, and cattle as a part of toxicokinetic and in vivo efficacy studies with mycotoxin detoxifying agents [55,56,57]. 

#### 2.4.4. Carry-Over

In the reported procedure, no carry-over was observed for AFG2 and AFM1. For AFB1, AFB2, and AFG1, limited carry-over (0.14–0.16%, 0.12–0.14%, and 0.11%, respectively) was observed in the first solvent sample that was analyzed after the highest calibrator sample and was further reduced to ≤0.03% in the second solvent sample. In the third solvent sample, no peaks of AFB1, AFB2, and AFG1 could be detected. Although the impact of this carry-over on method accuracy and precision is negligible, it is advised to analyze at least one solvent sample after the highest calibrator and/or QC samples.

#### 2.4.5. Selectivity

Method selectivity was evaluated by comparing extracted blank samples with spiked samples. Within 1 min of the elution zone of each analyte, no interfering peaks (S/N > 3) were detected, ensuring proper quantification in the different matrices.

#### 2.4.6. Stability 

The stability of AFs in sample extracts that were stored in the autosampler (8 °C) during at least nine days (cattle plasma) and 43 days (chicken plasma) shows that long analytical batches can be run, which makes the current method suitable for toxicokinetic and/or monitoring studies. Results of stability experiments further showed that the mean measured AFs concentrations fell within the accuracy acceptance criteria [58] after three freeze-thaw cycles and after a storage period of at least nine days (cattle plasma) or 63 days (chicken plasma) at ≤−15 °C.

The stability of AFs under different conditions was also shown by other authors: AFs in stock solutions were stable for at least 12 months in plastic tubes stored at −20 °C and for at least two months in working solutions stored at 4 °C [11]; AFB1 was stable for six weeks in working solutions stored at −20 °C [24], and AFB1 was stable in rat plasma and tissues during short-term (4 °C), long-term (−20 °C) and freeze-thaw conditions [27]; Corcuera et al. [24] reported that concentrations of AFB1 in spiked plasma and tissue samples were stable at −80 °C during six months. AFs were stable in human plasma extracts for 96 h [33], for 15 h in processed rat plasma, liver, and kidney [24], for up to 120 h in extracted milk samples [15] in the autosampler tray, and a maximum of 14 days in milk extracts stored at −20 °C [34]. During three freeze-thaw cycles, however, AFG1 and AFG2 proved not to be stable in plasma samples during bench stability experiments for 3 h and 6 h at room temperature [33]. This means that plasma should be processed immediately after thawing.

The obtained results indicated there were no problems with stability during the routine analysis of samples.

#### 2.4.7. Extraction Recovery and Matrix-Effects

LC-MS/MS is a very specific and selective analytical technique, but it has been demonstrated that co-eluting matrix interferences may have an impact on ionization efficiency [29,39,59]. This phenomenon can be limited by optimizing sample clean-up, chromatographic separation and by the use of isotope-labeled internal standards. To reduce the cost of the analysis, only three isotope-labeled ISs were used (^13^C_17_-AFB1, ^13^C_17_-AFG1, and ^13^C_17_-AFM1). Moreover, matrix-matched calibration curves were prepared in the present study to reduce further the influences of matrix effects on the UHPLC-MS/MS instrument. Matrix-matched calibration was also used by other authors [1,5,27,35,36] and demonstrated to be an effective way of reducing signal suppression or enhancement.

Extraction recoveries (R_E_) between 70 to 110% are generally accepted when analyzing food [35]. Acceptable R_E_ were obtained for cattle plasma (57.5–80.0%), cattle ruminal fluid (61.4–75.1%), chicken plasma (66.1–73.5%), and chicken muscle (114–142.5%). R_E_’s were rather low for cattle milk (24.7–47.8%), chicken liver (28.5–39.3%), and chicken eggs (7.5–23.9%) (see Tables S10 and S17). Results for matrix effects were acceptable for chicken plasma (60.2–88.5%), chicken muscle (61.4–89.0%), and cattle plasma (74.6–104.9%). Signal suppression was observed in chicken liver (28.0–79.1%), cattle milk (53.3–80.8%), and ruminal fluid (21.8–37.2%), whereas some signal enhancement was detected for chicken eggs (96.8–141.8%). However, by preparing matrix-matched calibration curves and using ^13^C-labelled internal standards, the impact of analyte loss during sample clean-up and matrix effects on the UHPLC-MS/MS instrument could be minimized, as has been shown during method validation. 

### 2.5. Analysis of Incurred Samples

To prove the applicability of the methods, incurred biological samples were analyzed that were collected from chickens (plasma, liver, muscle, and eggs) fed with a diet containing a high AFs concentration (target: 500 µg kg^−1^ feed); or from cows (plasma, ruminal fluid, and milk) fed either a control diet or a diet contaminated with AFB1 (inclusion rate: 788 µg/cow/day, equivalent to 69.7 µg/kg dry matter intake or DMI) alone or in combination with a selected mycotoxin binder (see Table S1). An overview of the mean concentrations of AFs in different chicken matrices after treatment with high AFB1 concentrations (target inclusion rate: 500 µg kg^−1^ feed) is shown in Table 4. Detailed results for the whole trial will be reported in an article by Ochieng et al. [56] that will be submitted in the near future.

As can be seen from Table 4, AFB1 concentrations were detected in plasma and liver and eggs with the highest concentrations observed in chicken liver. In muscle, no AFB1 was detected. No other AFs were determined at concentrations > LOQ in all chicken matrices.

In Table 5, the mean concentrations of AFs in different cattle matrices in a control group (Control) and after treatment with AFs (AF, inclusion rate: 788 µg/cow/day, equivalent to 69.7 µg/kg DMI), with a mycotoxin binder alone (BEN, bentonite; inclusion rate; 60 g/cow/day) or in combination with AFs (AF + BEN) are shown. Detailed results of the other groups and treatments were reported by Kemboi et al. [57]. 

As shown in Table 5, AFB1 and AFM1 concentrations above the LOQ were detected in the AF-supplemented group. AF concentrations decreased by the inclusion of a mycotoxin binder (bentonite) in the feed. The other AFs were only detected to a limited extent, and their observed concentrations were generally below the LOQ or LOD. 

In the current study, parent AFs (AFB1, AFB2, AFG1, and AFG2) and major phase-I hydroxylation metabolites (AFM1 and AFM2) were included, as these were identified by Jurišić et al. [60] as appropriate biomarkers of AFs exposure in chickens. The AFB1-N^7^-guanine adduct is assigned mainly as a urinary biomarker, and therefore, the determination of this metabolite was not relevant to the current study. AFB1 is considered a good biomarker of acute exposure, which is important when performing efficacy testing of mycotoxin detoxifiers and/or modifiers in short-term in vivo studies. 

Another metabolite, the AFB1-lysine adduct, which is considered an important biomarker of chronic exposure in serum or plasma, was not included in the current analysis methods because albumin/lysin adducts would have precipitated during the first step of the sample preparation procedure (consisting of liquid extraction with 1% FA in ACN) [22]. 

Phase-II biotransformation reactions of AFB1 consist mainly of glutathione conjugation. However, Vidal et al. [61] mentioned that not all species are able to produce these conjugates. Hence, the glutathione-conjugate was not included as a biomarker in this study. In addition, no de-conjugation step using *Helix pomatia* was performed during the sample preparation procedure since glucuronidation and/or sulfation have not been reported as main phase-II detoxification pathways for AFB1. 

The above results demonstrate that the developed methods can be applied not only for toxicokinetic studies with aflatoxins in chickens and cattle but also for residue determination, food safety monitoring, and efficacy testing of potential mycotoxin binders and/or modifiers.

## 3. Conclusions

Methods were developed and in-house validated for the quantitative determination of the aflatoxins AFB1, AFB2, AFG1, and AFG2 and their hydroxylated metabolites (AFM1 and AFM2) in chicken plasma, edible tissues (muscle and liver) and eggs; and cattle plasma, ruminal fluid, and milk. The novelty of the methods consisted of the optimization of high-throughput sample preparation procedures for each matrix to allow the processing of ≥96 samples per day. The sample preparation procedures were rather generic, but special attention was paid to the removal of PLs. Hence, these procedures can also be tested for the extraction of other mycotoxins in animal biological matrices, which can be a future application of the presented methods. Furthermore, chromatographic analysis was performed within 10 min, which allowed the analysis of a large number of samples in a 24-h period.

The different procedures were validated in accordance with European guidelines (linearity, accuracy, within-day and between-day precision, limit of quantification, limit of detection, specificity, and carry-over), and the results generally met the predefined acceptance criteria. 

As a proof-of-concept, the final methods were applied to successfully determine AFs concentrations in incurred chicken and cattle samples taken during a feeding trial to evaluate the efficacy and safety of mycotoxin detoxifying agents to protect against AFs as well as their carry-over to animal products.

## 4. Materials and Methods

### 4.1. Chemicals and Reagents

The standards of AFB1, AFB2, AFG1, AFG2, AFM1, and AFM2 were obtained from Fermentek Ltd. (Jerusalem, Israel) and had a certified purity of ≥ 98%. The internal standards (IS), ^13^C_17_-AFB1, ^13^C_17_-AFM1, and ^13^C_17_-AFG1, were purchased as 0.5 µg mL^−1^ solutions in ACN from Biopure (Tulln, Austria). All analytical standards were stored at the temperature suggested by the manufacturer. 

Methanol (MeOH), ACN, and formic acid (FA) were of ULC-MS grade and were obtained from Biosolve (Valkenswaard, The Netherlands). ULC-MS grade water was taken from a Milli-Q system (Merck, Overijse, Belgium). All other solvents and reagents had an analytical grade (formic acid, magnesium sulfate (MgSO_4_), sodium chloride (NaCl)) and were obtained from VWR (Leuven, Belgium). 

Oasis^®^ Ostro protein precipitation & phospholipid removal 96-well plates (25 mg), Oasis^®^ PRiME HLB 96-well plates (30 mg), 2 mL square 96-well collection plates and 96-well cap-mats with square plugs (silicone/PTFE treated, pre-slit) were purchased from Waters (Antwerp, Belgium).

### 4.2. Preparation of Standard Solutions

Standard stock solutions (SS) of AFB1 (1 mg mL^−1^), AFB2 (1 mg mL^−1^), AFG1 (1 mg mL^−1^), AFG2 (1 mg mL^−1^), AFM1 (0.1 mg mL^−1^) and AFM2 (0.1 mg mL^−1^) were prepared in ACN. An individual working solution (WS_ind_) with a concentration level of 100 µg mL^−1^ was prepared for AFB1, AFB2, AFG1, and AFG2 in ACN. Mixed working solutions of all aflatoxins (WS_mix_) at concentrations of 1000 ng mL^−1^, 100 ng mL^−1^, 10 ng mL^−1^, 1 ng mL^−1^, and 0.1 ng mL^−1^ were prepared by appropriate dilution of the SS and WS_ind_ in ACN. 

A mixed working solution (WS_IS_mix_) of the ISs, containing ^13^C_17_-AFB1, ^13^C_17_-AFM1, and ^13^C_17_-AFG1 at a concentration level of 10 ng mL^−1^ was prepared in ACN. 

All working solutions were kept at ≤−15 °C, protected from light, for at least six months.

### 4.3. Biological Samples

#### 4.3.1. Blank Samples

Blank plasma and tissue samples (liver and muscle) and eggs were obtained from broiler chickens or laying hens, and blank plasma, milk, and ruminal fluid samples were obtained from dairy cattle that received no AFs. These blank samples were used for the preparation of matrix-matched calibrator and quality control (QC) samples and were stored at ≤−15 °C.

#### 4.3.2. Incurred Samples

Incurred plasma, tissue (muscle and liver), and egg samples from chickens and plasma, milk, and ruminal fluid samples from cattle were analyzed.

Chicken plasma, tissues (liver, muscle), and egg samples were taken as a part of an in vivo efficacy and safety study with a mycotoxin detoxifying agent to reduce the carry-over of AFs to chicken products. Four hundred laying chickens (Isa Brown, 21 weeks old, 1.68 kg ± 0.1 kg) were bought from a commercial farm. The trial was conducted for four weeks, and samples (eggs, muscle, liver, and plasma) were collected at the end of the trial. More details concerning the animal experiments will be presented in a forthcoming article by Ochieng et al. [56]. The results of the analysis of layer chicken matrices for AFs, after feeding a diet containing AFB1 at a high target concentration of 500 µg kg^−1^ feed for four weeks, are shown in Table 4. All samples were stored at <−15 °C until sample mincing, homogenizing, and analysis. 

Dairy cattle plasma, ruminal fluid, and milk samples were taken as part of an in vivo efficacy and safety study with a mycotoxin detoxifier to reduce the carry-over of AFs to milk. Therefore, 24 animals (18 Borans and 6 Friesian-Boran crosses) in the early lactation (days in milk (mean ± SD) = 30.7 ± 5.7, body weight (mean ± SD) = 341.0 ± 33.8 kg) were used. The Borans were randomly divided into three groups of six animals each and assigned to one of three experiments, while the 6 crosses were only used for one experiment (for more details on the animal experiments, see Kemboi et al.) [57]. The Borans of which the results are shown in Table 5 were fed either an AFB1 contaminated diet (inclusion rate: 788 µg/cow/day, equivalent to 69.7 µg/kg dry matter intake or DMI) alone or a diet contaminated with AFB1 and supplemented with a selected mycotoxin binder. An overview of the different treatments is given in Supplementary Table S1. Plasma, milk, and ruminal fluid samples was taken at the start and end of the treatment period (2 weeks). All samples were kept at <−15 °C until sample analysis. 

More details concerning the animal experiments in cattle were described by Kemboi et al. [57].

### 4.4. Sample Pre-Treatment

#### 4.4.1. Chicken and Cattle Plasma

To 100 µL of chicken or cattle plasma were added 25 µL of the mixed IS working solution (WS_IS_mix_ 10 ng mL^−1^) and 100 µL of ACN, followed by vortex mixing and equilibration for 5 min at room temperature. After the addition of three hundred (300) µL of 1% FA in ACN, samples were vortex mixed (15 s) and centrifugated (10 min, 8517× *g*). The supernatant was applied to an Oasis^®^ Ostro 96-well plate (25 mg) and allowed to pass through the 96-well plate by vacuum application (15 mm Hg) for 5 min, and the filtrate was collected in a 96-well collector plate. After evaporation under a nitrogen stream (~40 °C), the dry residue was reconstituted in 200 µL of water/MeOH (50/50, v/v) and the 96-well collector plate was vortex mixed for 15 s. A mat cap was applied onto the 96-well collector plate, and a 5.0-µL aliquot of each sample was analyzed using the UHPLC-MS/MS instrument.

#### 4.4.2. Cattle Milk and Ruminal Fluid

To 1.0 mL of cattle milk or ruminal fluid was added 25 µL of the WS_IS_mix_ 10 ng mL^−1^, followed by vortex mixing and equilibration for 5 min at room temperature. Three mL of 1% FA in ACN were added, and after vortex mixing (30 s), the samples were extracted on a vertical rotary apparatus for 10 min (80 rpm), followed by a centrifugation step for 10 min at 1200× *g*. One (1) mL of the supernatant was applied to an Oasis PRiME HLB 96-well plate (30 mg of sorbent/well), and the filtrate was collected into a 2-mL 96-well square collector plate. The sample was evaporated to dryness under a nitrogen stream (~60 °C) and re-dissolved in 250 µL of water/MeOH (50/50, v/v). A 5.0-µL aliquot was injected into the UHPLC-MS/MS instrument. 

#### 4.4.3. Chicken Liver

To 1.0 g of chicken liver, 25 µL of the WS_IS_mix_ 10 ng mL^−1^ was added, followed by vortex mixing and equilibration for 5 min at room temperature. Liquid extraction was performed by the addition of three mL of 1% FA in ACN, followed by vortex mixing (5 min), extraction on a rotary apparatus for 10 min (80 rpm), and vortex mixing (5 min). The sample was centrifuged for 10 min at 1200× *g*, after which the supernatant was transferred to an extraction tube containing 0.2 g of sodium chloride (NaCl) and 0.8 g of magnesium sulfate (MgSO_4_). Next, the sample was vortex mixed again for 1 min, followed by a centrifugation step of 10 min at 1200× *g*. The supernatant was pipetted into another tube and evaporated to dryness under a nitrogen stream (~40 °C). Two hundred and fifty (250) µL of 1% FA in ACN were added to the dry residue, followed by vortex mixing for 15 s. The sample was transferred to an Oasis Ostro 96-well plate (25 mg) and allowed to pass through the 96-well plate by the application of a vacuum (15 mm Hg) for 10 min. The eluate was collected in a square 96-well collector plate and diluted with 250 µL of water, followed by gentle mixing (1000 rpm). The 96-well plate was covered with a cap mat, and a 5.0-µL aliquot was injected onto the UHPLC-MS/MS instrument. 

#### 4.4.4. Chicken Muscle

One (1.0) g of chicken muscle was transferred to a 15-mL extraction tube, and 25 µL of the WS_IS_mix_ 10 ng mL^−1^ was added, followed by vortex mixing and equilibration for 5 min at room temperature. Two (2) mL of water and 3 mL of 1% FA in ACN were added, each followed by a vortex mixing step of 2 min and 5 min, respectively, on a multi-tube vortex mixer. Extraction was performed for 15 min on a rotary apparatus (80 rpm), followed by a vortex mixing step (5 min) and centrifugation for 10 min at 1200× *g*. The supernatant was added to another extraction tube containing 0.2 g of NaCl and 0.8 g of MgSO_4_, followed by a 1-min vortex mixing step. After centrifugation (10 min at 1200× *g*), 500 µL of the supernatant was added to an autosampler vial and diluted by the addition of 200 µL of water, followed by vortex mixing (30 s). A 5.0-µL aliquot was analyzed using the UHPLC-MS/MS instrument. 

#### 4.4.5. Egg

One (1.0) g egg was weighed in a 15-mL extraction tube, and 25 µL of the WS_IS_mix_ 10 ng mL^−1^ was added, followed by vortex mixing and equilibration for 5 min at room temperature. Liquid extraction was performed using 3 mL of 1% FA in ACN, followed by vortex mixing (5 min) and extraction on a rotary apparatus for 10 min (80 rpm). After centrifugation for 10 min at 1200× *g*, the supernatant was added to another tube and evaporated under a gentle nitrogen stream (40 °C). To the dry residue, 250 µL of 1% FA in ACN was added, followed by vortex mixing for 15 s. Thereafter the sample was applied onto an Oasis Ostro 96-well plate (25 mg). The sample was allowed to pass through the 96-well plate by vacuum application (15 mm Hg) for 10 min, and the eluate was collected in a 96-well 2-mL collector plate. After the addition of 250 µL of water to each well and gentle mixing (30 s, 1000 rpm), the 96-well collector plated was covered with a cap mat, and a 5.0-µL aliquot was analyzed using the UHPLC-MS/MS instrument.

### 4.5. UHPLC-MS/MS Analysis for Quantification

The UHPLC system was an Acquity UPLC H-Class Quaternary Solvent Manager in combination with a Flow-Through-Needle Sample Manager with temperature controlled tray and column oven from Waters. An Acquity UPLC HSS T3 column (100 mm × 2.1 mm i.d., dp: 1.8 µm) in combination with an Acquity HSS T3 1.8 μm Vanguard pre-column, both from Waters, was used for chromatographic separation. 

The mobile phase A was water, while the mobile phase B was MeOH. The following gradient was used: 0–1.0 min (80% A, 20% B), 3.0 min (linear gradient to 90% B), 3.0–7.0 min (10% A, 90% B), 7.3 min (linear gradient to 80% A), 7.3–10.0 min (80% A, 20% B). The flow rate was 0.3 mL min^−1^.

The column oven and autosampler tray were kept at 40 °C and 8 °C, respectively. 

The UHPLC column effluent was transferred to a Xevo TQ-S^®^ MS/MS system with an electrospray ionization (ESI) probe that operated in the positive ionization mode (all from Waters). The divert valve was programmed to send the UPLC effluent to the mass spectrometer between 3.0 and 6.0 min.

Mass spectrometric parameters were determined by infusing working solutions of 100 ng mL^−1^ of all aflatoxins and the ISs at a flow rate of 10 µL min^−1^ in combination with the mobile phase (50% A, 50% B, flow rate: 200 µL min^−1^). 

The following parameters were selected: capillary voltage: 3.2 kV, source offset: 50 V, source temperature: 150 °C, desolvation temperature: 600 °C, desolvation gas: 800 L h^−1^, cone gas: 150 L h^−1^, nebulizer pressure: 6.9 bar, LM resolution 1 and 2: 2.8, HM resolution 1 and 2: 15, ion energy 1 and 2: 0.2 and 0.8, respectively, collision gas flow: 0.15 mL min^−1^. 

MS/MS data acquisition was performed in the multiple reaction monitoring (MRM) mode. The MRM transitions that were measured for all analytes are shown in Table 1.

### 4.6. Method Validation

The UHPLC-MS/MS method was validated in-house for the analytes of interest (AFB1, AFB2, AFG1, AFG2, AFM1, and AFM2) by analyzing spiked blank biological samples obtained from untreated chickens and cattle. The following parameters were evaluated as recommended by European guidelines and in accordance with the literature [32,58,62,63,64,65]: linearity, within- and between-run accuracy and precision, the limit of quantification (LOQ), the limit of detection (LOD), carry-over, freeze-thaw stability, matrix effect (M_E_) and extraction recovery (R_E_). 

#### 4.6.1. Calibration Curves

For the preparation of matrix-matched calibration curves, 100 µL of blank chicken and cattle plasma (concentration range: LOQ–200 ng mL^−1^), 1 g of blank chicken tissues (liver and muscle) and eggs, and 1 mL of blank cattle milk or ruminal fluid (concentration range: LOQ–10 ng mL^−1^ or µg kg^−1^) were used. Three different calibration curves were extracted on three different analysis days. The correlation coefficients (r) and goodness-of-fit coefficients (gof) were evaluated and had to comply with the acceptance criteria for r ≥ 0.99 and g ≤ 20%, respectively [32,62]. The most appropriate weighting factor (1/x^0^, 1/x^1^, 1/x^2^) was selected based on VICH GL49 guidelines [58]. The procedure was as follows: the calibration curve was constructed using three weighting factors, and the gof’s were calculated. The gof’s of the three individual curves were summed per weighting factor; the weighting factor that gave the smallest sum of gof was selected. In addition, the calculated concentration of the individual calibrator samples had to fall within the ranges for accuracy at the specified level. 

#### 4.6.2. Accuracy and Precision 

To determine within-run accuracy and precision (repeatability), six blank samples that were spiked at a low (LOQ), medium, and high concentration level were analyzed in the same run. For the evaluation of the between-run accuracy and precision (reproducibility), at least three blank samples spiked at the same concentration levels were analyzed on three different days. The results had to fulfill acceptance criteria for accuracy as described in VICH GL49. The relative standard deviation (RSD) was calculated and had to be below the RSD_max_ value [58]. 

#### 4.6.3. Limit of Quantification and Limit of Detection

The LOQ was defined as the lowest concentration of the analyte for which the method was validated with an accuracy and precision that fell within the specified ranges. The LOQ was also set as the lowest point of the calibration curve. To determine the LOQ, six samples spiked at a concentration level between 0.025 and 0.50 ng mL^−1^ or µg kg^−1^ were analyzed on the same day [58]. 

The lowest concentration that could be recognized by the detector with a signal-to-noise (S/N) ratio of ≥ 3 was defined as the LOD. The LOD values were calculated using the mean S/N of the blank samples spiked at the LOQ level [58].

#### 4.6.4. Carry-Over

The reconstitution solvent was injected after the highest calibration sample to evaluate the presence or absence of carry-over. If a peak was observed in the elution zone of an analyte or the IS, it had to be below 20% of the LOQ and below 5% for the IS [65].

#### 4.6.5. Extraction Recovery, Matrix-Effects, and Process Efficiency

To assess extraction recovery (R_E_) and matrix effects (M_E_) quantitatively, three sets of samples were prepared: set A consisted of standard solutions of the analytes of interest (concentration: 0.50 and 5.0 ng mL^−1^ or µg kg^−1^); the two other sets consisted of matrix-matched samples that were spiked with AFs after (set B) and before (set C) extraction at the same concentration levels as the set-A samples. The R_E_ and M_E_ (%) were calculated by dividing the peak areas of AFs in the respective samples, i.e., R_E_ = C/B × 100 and M_E_ = B/A × 100.

#### 4.6.6. Storage Stability

The stability of AFs was evaluated in extracted samples (QC_stab_extr_) in the matrix during storage at ≤−15 °C (QC_stab_matrix_) and during three freeze-thaw cycles (QC_freeze_thaw_). For each stability experiment, blank samples were spiked with AFs at a concentration of 0.50 and 5.0 ng mL^−1^ or µg kg^−1^. 

*Stability in extracted samples.* Stability in extracted samples was assessed because samples are sometimes processed one day and assayed on a second day or are stored for additional days due to an instrument failure, e.g., over a weekend. The QC_stab_extr_ samples (n = 3 per concentration level) were extracted as specified above (Section 4.4) and stored at 2–8 °C, which corresponds with autosampler and/or refrigerator temperature. After a specified storage period, the QC_stab_extr_ samples were analyzed on the UHPLC-MS/MS instrument and quantified using a freshly prepared matrix-matched calibration curve. 

*Freeze-thaw stability.* The QC_freeze_thaw_ samples (n = 3 per concentration level) were frozen and stored in the freezer at ≤−15 °C and thawed at room temperature. During each cycle, samples were frozen for at least 12 h before they were thawed [65]. After three freeze-thaw cycles, the QC_freeze_thaw_ samples were extracted and analyzed on the UHPLC-MS/MS instrument. Quantification was performed using a freshly prepared matrix-matched calibration curve.

*Stability in matrix.* The QC_stab_matrix_ samples were put in the freezer at ≤−15 °C immediately after preparation. After a certain storage period, the samples were thawed and analyzed using the above described sample preparation procedure and UHPLC-MS/MS method. Samples were quantified using a freshly prepared matrix-matched calibration curve.

QC_stab_extr_, QC_freeze_thaw_, and QC_stab_matrix_ sample stability were considered acceptable if the mean concentration determined at the specified stability time point agreed with the spiked theoretical concentrations within the accuracy acceptance criteria [58,65].

## Figures and Tables

**Table 1:** MRM transitions and MS/MS parameters for the aflatoxins under investigation (UHPLC-MS/MS analysis).

Analyte	Chemical Formula	MM ^a^ (g mol^−1^)	Precursor Ion (*m/z*) ^b^	Product Ions (*m/z*)	CE ^c^ (eV)	Cone (V)	Retention Time (Min)	Internal Standard
AFB1	C_17_H_12_O_6_	312.27	313.0 [M + H]^+^	241.1 ^d^ 285.1	34 23	35 35	4.55	^13^C_17_-AFB1
AFB2	C_17_H_14_O_6_	314.29	315.1 [M + H]^+^	259.1 ^d^ 287.1	25 24	30 30	4.48	^13^C_17_-AFB1
AFG1	C_17_H_12_O_7_	328.27	329.1 [M + H]^+^	243.0 ^d^ 311.1	23 18	35 35	4.38	^13^C_17_-AFG1
AFG2	C_17_H_14_O_7_	330.29	331.1 [M + H]^+^	245.0 ^d^ 313.1	26 22	30 30	4.30	^13^C_17_-AFB1
AFM1	C_17_H_12_O_7_	328.27	328.9 [M + H]^+^	229.0 272.9 ^d^	35 20	30 30	4.31	^13^C_17_-AFM1
AFM2	C_17_H_14_O_7_	330.29	331.0 [M + H]^+^	285.0 313.1 ^d^	21 15	35 35	4.22	^13^C_17_-AFM1
^13^C_17_-AFB1	^13^C_17_H_12_O_6_	329.15	330.1 [M + H]^+^	255.1 ^d^ 301.0	35 28	20 20	4.55	/
^13^C_17_-AFM1	^13^C_17_H_12_O_7_	345.15	346.1 [M + H]^+^	273.0 288.1 ^d^	25 25	30 30	4.31	/
^13^C_17_-AFG1	^13^C_17_H_12_O_7_	345.15	346.1 [M + H]^+^	257.1 ^d^ 328.1	25 20	40 40	4.38	/

^a^ MM = molecular mass, ^b^ *m/z* = mass to charge ratio, ^c^ CE = collision energy, ^d^ ion used for quantification.

**Table 2:** Results of the evaluation of linearity (slope (a), intercept (b), goodness-of-fit coefficient (gof), correlation coefficient (r)), the limit of quantification (LOQ), the limit of detection (LOD) for aflatoxins in chicken plasma.

Component	Calibration Range (ng mL^−1^)	a	b	gof (%)	r	LOQ (ng mL^−1^)	LOD (ng mL^−1^)
AFB1	0.050–200.0	0.852 ± 0.018	0.0002 ± 0.0025	5.7 ± 2.5	0.9978 ± 0.0019	0.050	0.0029
AFB2	0.050–200.0	0.957 ± 0.014	0.0063 ± 0.0122	6.5 ± 2.7	0.9972 ± 0.0022	0.050	0.0035
AFG1	0.050–200.0	0.408 ± 0.001	0.0088 ± 0.0135	5.8 ± 0.8	0.9980 ± 0.0006	0.050	0.0069
AFG2	0.050–200.0	0.159 ± 0.010	−0.0022 ± 0.0030	6.8 ± 1.4	0.9973 ± 0.0010	0.050	0.0193
AFM1	0.050–200.0	0.540 ± 0.043	−0.0015 ± 0.0026	6.6 ± 1.1	0.9975 ± 0.0009	0.050	0.0035
AFM2	0.50–200	0.147 ± 0.026	0.0063 ± 0.0066	4.8 ± 13	0.9984 ± 0.0008	0.100	0.0300

**Table 3:** Results of the within-run and between-run precision and accuracy evaluation for the analysis of aflatoxins in chicken plasma.

Component	Theoretical Concentration (ng mL^−1^)	Mean Concentration ± SD (ng mL^−1^)	Precision, RSD (%)	Accuracy (%)
AFB1	0.050 ^a^	0.040 ± 0.004	10.2	−20.9
	0.050 ^b^	0.044 ± 0.006	12.7	−11.6
	0.50 ^a^	0.49 ± 0.02	3.8	−1.6
	0.50 ^b^	0.51 ± 0.05	10.1	1.9
	5.00 ^a^	5.26 ± 0.12	2.3	5.1
	5.00 ^b^	5.10 ± 0.33	6.4	2.0
	50.0 ^a^	44.0 ± 1.4	3.2	−12.1
	50.0 ^b^	45.9 ± 3.3	7.2	−8.1
AFB2	0.050 ^a^	0.056 ± 0.005	8.7	10.9
	0.050 ^b^	0.041 ± 0.008	18.6	−17.9
	0.50 ^a^	0.54 ± 0.02	3.9	7.5
	0.50 ^b^	0.50 ± 0.07	13.3	0.5
	5.00 ^a^	5.32 ± 0.25	4.7	6.4
	5.00 ^b^	4.95 ± 0.43	8.7	−1.1
	50.0 ^a^	50.1 ± 1.2	2.4	0.2
	50.0 ^b^	50.6 ± 3.5	6.9	1.2
AFG1	0.050 ^a^	0.054 ± 0.009	16.3	7.3
	0.050 ^b^	0.051 ± 0.015	30.4	1.8
	0.50 ^a^	0.50 ± 0.01	2.1	−0.6
	0.50 ^b^	0.53 ± 0.07	13.0	6.2
	5.00 ^a^	4.88 ± 0.14	3.0	−2.4
	5.00 ^b^	5.20 ± 0.68	13.0	4.1
	50.0 ^a^	48.2 ± 1.8	3.7	−3.6
	50.0 ^b^	48.3 ± 2.2	4.5	−3.4
AFG2	0.050 ^a^	0.037 ± 0.008	22.1	−25.2
	0.050 ^b^	0.048 ± 0.015	30.2	−3.7
	0.50 ^a^	0.48 ± 0.01	3.1	−4.9
	0.50 ^b^	0.51 ± 0.10	20.4	1.4
	5.00 ^a^	4.54 ± 0.31	6.8	−9.2
	5.00 ^b^	4.98 ± 0.40	8.0	−0.4
	50.0 ^a^	51.8 ± 2.5	4.8	3.6
	50.0 ^b^	50.8 ± 3.4	6.8	1.5
AFM1	0.050 ^a^	0.055 ± 0.003	6.2	9.0
	0.050 ^b^	0.042 ± 0.008	19.4	−15.6
	0.50 ^a^	0.51 ± 0.01	2.3	1.3
	0.50 ^b^	0.52 ± 0.10	18.5	4.4
	5.00 ^a^	4.95 ± 0.16	3.2	−1.0
	5.00 ^b^	5.04 ± 0.31	6.1	0.8
	50.0 ^a^	49.6 ± 0.9	1.7	−0.9
	50.0 ^b^	50.3 ± 6.4	12.8	0.7
AFM2	0.50 ^a^	0.55 ± 0.06	11.3	9.1
	0.50 ^b^	0.49 ± 0.06	12.0	−1.7
	5.00 ^a^	4.61 ± 0.10	2.2	−7.8
	5.00 ^b^	4.75 ± 0.15	3.2	−5.0

Note: ^a^ Within-run accuracy and precision (n ≥ 5); ^b^ Between-run accuracy and precision (n ≥ 3 × 6); SD: standard deviation; RSD: relative standard deviation; Acceptance criteria: accuracy: <1 ng mL^−1^: −50% to +20%, ≥1 to <10 ng mL^−1^: −40% to +20%, ≥10 to <100 ng mL^−1^: −30% to +10%, ≥100 ng mL^−1^: −20% to +10%; within-run precision (RSD_max_): <1 ng mL^−1^: 30%, ≥1 to <10 ng mL^−1^: 25%, ≥10 to <100 ng mL^−1^: 15%, ≥100 ng mL^−1^: 10%; between-run precision: <1 ng mL^−1^: 45%, ≥1 to <10 ng mL^−1^: 32%, ≥10 to <100 ng mL^−1^: 23%, ≥100 ng mL^−1^: 16% [58].

**Table 4:** Mean (± standard deviation) aflatoxins concentrations in the different layer chicken matrices (plasma, liver, muscle, and eggs) taken after treatment with high AFs concentrations (target inclusion rate: 500 µg kg^−1^ feed).

Matrix	AFB1	AFB2	AFG1	AFG2	AFM1	AFM2
	Analyte Concentration (µg kg^−1^)					
Plasma (n = 8)	0.063 ± 0.073	ND	ND	ND	<LOQ	ND
Liver (n = 8)	0.474 ± 0.210	ND	<LOD	<LOD	<LOQ	<LOQ
Muscle (n = 8)	ND	ND	ND	ND	ND	ND
Egg (n = 8)	0.039 ± 0.018	<LOD	<LOD	ND	<LOQ	<LOD

Note: ND = not detected, i.e., no analyte peaks are determined; <LOQ = below limit of quantification; <LOD = below limit of detection; AFB1 = aflatoxin B1; AFB2 = aflatoxin B2; AFG1 = aflatoxin G1; AFG2 = aflatoxin G2; AFM1 = aflatoxin M1; AFM2 = aflatoxin M2.

**Table 5:** Mean (± standard deviation, n = 6) aflatoxins concentrations in the different dairy cattle matrices (plasma, milk, and rumen fluid) after treatment with a control diet (control), a diet containing only a mycotoxin binder (BEN) or a diet containing AFs (inclusion rate: 788 µg/cow/day, equivalent to 69.7 µg/kg DMI) alone (AF) or in combination with a binder (AF + BEN).

Matrix	Component	Treatment Group			
		Control	AF	BEN	AF + BEN
		Analyte Concentration (µg kg^−1^)			
Milk	AFB1	ND	0.068 ± 0.038	ND	<LOQ
	AFB2	ND	<LOQ	ND	<LOQ
	AFM1	0.039 ± 0.025	0.862 ± 0.406	<LOQ	0.602 ± 0.309
	AFM2	<LOQ	ND	ND	<LOD
	AFG1	ND	ND	ND	<LOQ
	AFG2	ND	ND	ND	<LOQ
Plasma	AFB1	<LOD	0.028 ± 0.014	<LOQ	<LOQ
	AFB2	ND	ND	ND	ND
	AFM1	<LOQ	0.040 ± 0.018	<LOQ	0.026 ± 0.022
	AFM2	ND	ND	ND	ND
	AFG1	<LOQ	<LOQ	<LOQ	<LOQ
	AFG2	ND	ND	ND	ND
Rumen fluid	AFB1	<LOD	0.337 ± 0.177	<LOD	0.369 ± 0.265
	AFB2	<LOD	<LOQ	<LOD	<LOQ
	AFM1	ND	<LOQ	ND	<LOQ
	AFM2	<LOD	ND	<LOD	ND
	AFG1	ND	ND	ND	ND
	AFG2	ND	ND	ND	ND

Note: Control; measurement before treatment; ND = not detected; <LOQ = below limit of quantification; <LOD = below limit of detection.

## Data Availability

Data is contained within the article or supplementary material and in the articles of Ochieng et al [55,56] and Kemboi et al. [57].

## Supplementary Materials

The following supporting information can be downloaded at: https://www.mdpi.com/article/10.3390/toxins15010037/s1, Table S1. Overview of the different treatments that were administrated for two weeks to dairy cattle during an in vivo efficacy and safety study with mycotoxin detoxifying agents. Table S2. Results of the evaluation of linearity (slope (a), intercept (b), goodness-of-fit coefficient (gof), correlation coefficient (r)), the limit of quantification (LOQ), the limit of detection (LOD) for aflatoxins in cattle plasma, milk and ruminal fluid. Table S3. Results of the evaluation of linearity (slope (a), intercept (b), goodness-of-fit coefficient (gof), correlation coefficient (r)), the limit of quantification (LOQ), the limit of detection (LOD) for aflatoxins in chicken liver, muscle, and eggs. Table S4. Results of the within-run and between-run precision and accuracy evaluation for the analysis of aflatoxins in cattle plasma. Table S5. Results of the stability evaluation of aflatoxins in cattle plasma sample extracts (stored at 8 °C) in cattle plasma during three freeze-thaw cycles and storage at ≤−15 °C. Table S6. Results of the within-run and between-run precision and accuracy evaluation for the analysis of aflatoxins in cattle milk. Table S7. Results of the stability evaluation of aflatoxins in milk sample extracts (stored at 2–8 °C) and milk during three freeze-thaw cycles and storage at ≤−15 °C. Table S8. Results of the within-run and between-run precision and accuracy evaluation for the analysis of aflatoxins in cattle ruminal fluid. Table S9. Results of the stability evaluation of aflatoxins in ruminal fluid sample extracts (stored at 8 °C) and in ruminal fluid during three freeze-thaw cycles. Table S10. Results of the evaluation of extraction recovery and matrix effect for the UHPLC-MS/MS analysis of aflatoxins in cattle plasma, milk, and ruminal fluid. Table S11. Results of the within-run and between-run precision and accuracy evaluation for the analysis of aflatoxins in chicken liver. Table S12. Results of the stability evaluation of aflatoxins in chicken liver sample extracts (stored at 8 °C) in the chicken liver during three freeze-thaw cycles and storage at ≤−15 °C. Table S13. Results of the within-run and between-run precision and accuracy evaluation for the analysis of aflatoxins in chicken muscle. Table S14. Results of the stability evaluation of aflatoxins in chicken muscle sample extracts (stored at 8 °C) in chicken muscle during three freeze-thaw cycles and storage at ≤−15 °C. Table S15. Results of the within-run and between-run precision and accuracy evaluation for the analysis of aflatoxins in eggs. Table S16. Results of the stability evaluation of aflatoxins in egg sample extracts (stored at 8 °C), in eggs during three freeze-thaw cycles, and during storage at ≤−15 °C. Table S17. Results of the evaluation of extraction recovery and matrix effect for the UHPLC-MS/MS analysis of aflatoxins in chicken plasma, liver, muscle, and eggs. Figure S1. Evaluation of signal intensity (based on peak area) of AFB1, AFB2, AFG1, AFG2, AFM1, and AFM2 during chromatography with different aqueous mobile phases (MF), i.e. MF-1: water; MF-2: 5 mM NH4FA + 0.1% FA in water; MF-3: 10 mM NH4FA + 0.3% FA in water; MF-4: 5 mM NH4AA + 0.1% AA in water. Methanol was used as an organic mobile phase in all experiments.
