# Supplementary material for: Development of High-Throughput Sample Preparation Procedures for the Quantitative Determination of Aflatoxins in Biological Matrices of Chickens and Cattle Using UHPLC-MS/MS
Source: Toxins (Basel). 2023 Jan 3;15(1):37. doi: 10.3390/toxins15010037 (PMC9866995; doi:10.3390/toxins15010037)
Supplement: Supplementary file 1 [file toxins-15-00037-s001.zip › toxins-2080169-supplementary.pdf]

Development of High-Throughput Sample Preparation Procedures for the Quantitative Determination of Aflatoxins in Biological Matrices of Chickens and Cattle Using UHPLC-MS/MS

Siegrid De Baere, Phillis E. Ochieng, David C. Kemboi, Marie-Louise Scippo, Sheila Okoth, Johanna F. Lindahl, James K. Gathumbi, Gunther Antonissen and Siska Croubels

Table S1. Overview of the different treatments that were administrated for 2 weeks to dairy cattle during an *in vivo* efficacy and safety study with mycotoxin detoxifying agents.

Treatment N°	AFB1 contamination (788 µg/cow/day, equivalent to 69.7 µg/kg DMI)	Mycofix (60g/cow/day)
T1	/	/
T2	x	/
T3	/	x
T4	x	x
T5	/	/

Table S2. Results of the evaluation of linearity (slope (a), intercept (b), goodness-of-fit coefficient (gof), correlation coefficient (r)), limit of quantification (LOQ), limit of detection (LOD) for aflatoxins in cattle plasma, milk and ruminal fluid.

Component	Calibration Range (ng mL^{-1})	a	b	gof (%)	r	LOQ (ng mL^{-1})	LOD[1] (ng mL^{-1})
**plasma**							
AFB1	0.025 – 10.0	0.807 ± 0.035	-0.0001 ± 0.0034	4.5 ± 1.3	0.9986 ± 0.0008	0.025	0.002
AFB2	0.050 – 10.0	1.084 ± 0.258	0.0056 ± 0.0131	6.7 ± 2.9	0.9967 ± 0.0026	0.050	0.002
AFG1	0.050 – 10.0	0.450 ± 0.078	0.0063 ± 0.0020	6.7 ± 0.1	0.9971 ± 0.0001	0.050	0.005
AFG2	0.050 – 10.0	0.154 ± 0.005	-0.0001 ± 0.0023	7.7 ± 2.2	0.9960 ± 0.0020	0.050	0.009
AFM1	0.025 – 10.0	0.618 ± 0.063	0.0015 ± 0.0005	6.4 ± 1.6	0.9972 ± 0.0013	0.025	0.002
AFM2	0.50 – 10.0	0.221 ± 0.099	-0.0207 ± 0.0374	5.7 ± 5.4	0.9956 ± 0.0066	0.50	0.060
**Milk**							
AFB1	0.050 – 10.0	7.29 ± 0.06	0.0988 ± 0.0760	3.9 ± 0.7	0.9990 ± 0.0004	0.050	0.002
AFB2	0.050 – 10.0	16.48 ± 3.61	0.2041 ± 0.1450	5.3 ± 1.0	0.9981 ± 0.0008	0.050	0.002
AFG1	0.050 – 10.0	7.61 ± 2.58	0.0984 ± 0.0486	5.4 ± 0.3	0.9980 ± 0.0002	0.050	0.005
AFG2	0.050 – 10.0	2.19 ± 0.11	0.0409 ± 0.0258	4.6 ± 0.4	0.9985 ± 0.0002	0.050	0.005
AFM1	0.025 – 10.0	5.65 ± 0.43	0.0399 ± 0.0423	3.4 ± 1.6	0.9992 ± 0.0006	0.025	0.003
AFM2	0.50 – 10.0	0.96 ± 0.12	0.0415 ± 0.0266	3.7 ± 0.1	0.9988 ± 0.0000	0.50	0.038
**Ruminal fluid**							
AFB1	0.1 – 10.0	12.41 ± 4.91	-0.0289 ± 0.1838	6.5 ± 1.3	0.9970 ± 0.0011	0.10	0.027
AFB2	0.1 – 10.0	8.64 ± 4.67	0.1688 ± 0.9704	4.1 ± 1.7	0.9987 ± 0.0008	0.10	0.024
AFG1	0.1 – 10.0	3.70 ± 0.89	0.0239 ± 0.0676	6.7 ± 2.3	0.9967 ± 0.0023	0.10	0.025
AFG2	0.1 – 10.0	7.21 ± 3.56	0.4597 ± 0.5060	6.1 ± 3.2	0.9970 ± 0.0030	0.10	0.032
AFM1	0.1 – 10.0	9.30 ± 5.22	-0.0275 ± 0.1213	5.4 ± 0.5	0.9980 ± 0.0004	0.10	0.012
AFM2	0.5 – 10.0	2.18 ± 1.11	0.0819 ± 0.1845	4.4 ± 2.2	0.9981 ± 0.0018	0.50	0.132

Note: [1]LOD: calculated based on S/N = 3.

Table S3. Results of the evaluation of linearity (slope (a), intercept (b), goodness-of-fit coefficient (gof), correlation coefficient (r)), limit of quantification (LOQ), limit of detection (LOD) for aflatoxins in chicken liver, muscle and eggs.

Component	Calibration Range (μg kg^{-1})	a	b	gof (%)	r	LOQ (μg kg^{-1})	LOD[1] (μg kg^{-1})
**liver**							
AFB1	0.050 – 10.0	6.91 ± 0.14	0.14 ± 0.07	5.1 ± 0.4	0.9983 ± 0.0003	0.050	0.007
AFB2	0.10 – 10.0	13.43 ± 0.66	0.04 ± 0.39	6.3 ± 1.9	0.9971 ± 0.0017	0.10	0.006
AFG1	0.25 – 10.0	7.93 ± 1.24	0.02 ± 0.25	4.4 ± 2.2	0.9982 ± 0.0016	0.25	0.007
AFG2	0.25 – 10.0	2.70 ± 0.57	0.02 ± 0.01	2.8 ± 1.2	0.9993 ± 0.0005	0.25	0.010
AFM1	0.10 – 10.0	5.65 ± 0.03	0.15 ± 0.15	3.1 ± 0.5	0.9993 ± 0.0002	0.10	0.006
AFM2	0.50 – 10.0	2.73 ± 0.33	1.01 ± 1.17	6.5 ± 3.6	0.9957 ± 0.0040	0.50	0.040
**muscle**							
AFB1	0.050 – 10.0	7.59 ± 3.45	0.09 ± 0.15	6.2 ± 0.4	0.9976 ± 0.0005	0.050	0.013
AFB2	0.25 – 10.0	2.91 ± 0.77	-0.04 ± 0.05	6.1 ± 0.2	0.9971 ± 0.0002	0.25	0.028
AFG1	0.25 – 10.0	7.90 ± 2.67	0.11 ± 0.17	5.4 ± 1.7	0.9976 ± 0.0015	0.25	0.016
AFG2	0.25 – 10.0	2.75 ± 0.68	0.05 ± 0.12	5.2 ± 1.6	0.9977 ± 0.0013	0.25	0.039
AFM1	0.10 – 10.0	6.92 ± 1.11	-0.01 ± 0.10	4.8 ± 0.8	0.9985 ± 0.0004	0.10	0.014
**eggs**							
AFB1	0.025 – 10.0	14.43 ± 4.02	0.04 ± 0.09	5.6 ± 1.4	0.9979 ± 0.0009	0.025	0.003
AFB2	0.025 – 10.0	6.60 ± 1.80	0.01 ± 0.02	6.1 ± 1.7	0.9974 ± 0.0012	0.025	0.005
AFG1	0.050 – 10.0	4.13 ± 0.17	0.06 ± 0.03	4.9 ± 2.5	0.9981 ± 0.0015	0.050	0.008
AFG2	0.050 – 10.0	5.09 ± 1.62	0.02 ± 0.01	6.9 ± 2.8	0.9965 ± 0.0024	0.050	0.007
AFM1	0.025 – 10.0	12.98 ± 4.98	0.03 ± 0.04	4.3 ± 2.0	0.9986 ± 0.0010	0.025	0.002
AFM2	0.50 – 10.0	1.60 ± 0.89	0.22 ± 0.50	6.6 ± 2.1	0.9961 ± 0.0022	0.50	0.097

Note: [1]LOD: calculated based on S/N = 3.

Table S4. Results of the within-run and between-run precision and accuracy evaluation for the analysis of aflatoxins in cattle plasma.

Component	Theoretical concentration (ng mL^{-1})	Mean concentration ± SD (ng mL^{-1})	Precision, RSD (%)	Accuracy (%)
AFB1	0.025 [a]	0.028 ± 0.003	10.0	13.8
	0.025 [b]	0.024 ± 0.006	23.2	-5.1
	0.050 [a]	0.043 ± 0.003	7.9	-13.5
	0.050 [b]	0.047 ± 0.006	13.2	-6.2
	0.50 [a]	0.50 ± 0.02	3.3	-0.2
	0.50 [b]	0.49 ± 0.02	4.8	-1.2
	5.00 [a]	5.26 ± 0.17	3.3	5.1
	5.00 [b]	5.04 ± 0.21	4.1	0.9
AFB2	0.050 [a]	0.048 ± 0.005	10.9	-3.3
	0.050 [b]	0.050 ± 0.008	15.8	-0.7
	0.50 [a]	0.55 ± 0.02	3.2	9.2
	0.50 [b]	0.51 ± 0.05	8.8	1.7
	5.00 [a]	5.49 ± 0.11	2.0	9.7
	5.00 [b]	4.76 ± 0.55	11.6	-4.9
AFG1	0.050 [a]	0.055 ± 0.009	16.1	9.3
	0.050 [b]	0.047 ± 0.013	28.2	-5.6
	0.50 [a]	0.48 ± 0.04	7.4	-3.5
	0.50 [b]	0.49 ± 0.03	6.2	-1.4
	5.00 [a]	5.35 ± 0.32	6.0	7.0
	5.00 [b]	4.92 ± 0.38	7.7	-1.6
AFG2	0.050 [a]	0.054 ± 0.008	14.2	7.1
	0.050 [b]	0.052 ± 0.008	16.2	4.7
	0.50 [a]	0.47 ± 0.02	4.0	-5.8
	0.50 [b]	0.51 ± 0.04	8.5	2.1
	5.00 [a]	5.15 ± 0.50	9.6	2.9
	5.00 [b]	5.01 ± 0.32	6.4	0.1
AFM1	0.025 [a]	0.025 ± 0.002	6.6	-1.7
	0.025 [b]	0.027 ± 0.004	12.9	9.7
	0.050 [a]	0.051 ± 0.005	9.0	1.8
	0.050 [b]	0.053 ± 0.005	9.9	5.2
	0.50 [a]	0.55 ± 0.03	4.8	10.3
	0.50 [b]	0.53 ± 0.03	5.9	2.7
	5.00 [a]	5.20 ± 0.11	2.0	4.0
	5.00 [b]	5.19 ± 0.09	1.7	3.9
AFM2	0.50 [a]	0.48 ± 0.05	9.3	-3.4
	0.50 [b]	0.50 ± 0.06	11.5	0.3
	5.00 [a]	4.82 ± 0.09	1.8	-3.5
	5.00 [b]	4.75 ± 0.22	4.6	-5.0

Note: [a] Within-run accuracy and precision (n=6); [b] Between-run accuracy and precision (n= 3 x 6); SD: standard deviation; RSD: relative standard deviation; Acceptance criteria: accuracy: < 1 ng mL^{-1}: -50% to +20%, ≥ 1 to < 10 ng mL^{-1}: -40% to +20%, ≥ 10 to < 100 ng mL^{-1}: -30% to +20%; within-run precision (RSD_{max}): < 1 ng mL^{-1}: 30 %, ≥1 to < 10 ng mL^{-1}: 25.0%, ≥ 10 to < 100 ng mL^{-1}: 15%; between-run precision: < 1 ng mL^{-1}: 45%, ≥1 to < 10 ng mL^{-1}: 32%, ≥ 10 to < 100 ng mL^{-1}: 23% [VICH GL49].

Table S5. Results of the stability evaluation of aflatoxins in cattle plasma sample extracts (stored at 8 °C), in cattle plasma during 3 freeze-thaw cycles and during storage at ≤- 15 °C.

Storage conditions	Component	Theoretical concentration (ng mL^{-1})	Mean concentration ± SD (ng mL^{-1})	Accuracy (%)
Stability in extract	AFB1	0.50	0.52 ± 0.01	4.8
8 °C		5.00	5.42 ± 0.14	8.4
≥ 9 days	AFB2	0.50	0.50 ± 0.07	0.1
(n = 3)		5.00	5.17 ± 0.05	3.5
	AFG1	0.50	0.49 ± 0.09	-2.5
		5.00	4.94 ± 0.40	-1.2
	AFG2	0.50	0.53 ± 0.01	5.9
		5.00	5.70 ± 0.22	14.1
	AFM1	0.50	0.48 ± 0.01	-3.9
		5.00	4.62 ± 0.13	-7.6
	AFM2	0.50	0.33 ± 0.07	-33.7
		5.00	3.77 ± 0.19	-24.5
Freeze-thaw stability	AFB1	0.50	0.39 ± 0.01	-22.2
≤- 15 °C to room temperature		5.00	4.55 ± 0.05	-9.1
3 cycles	AFB2	0.50	0.53 ± 0.02	5.3
(n = 3)		5.00	4.71 ± 0.14	-5.7
	AFG1	0.50	0.46 ± 0.02	-8.2
		5.00	4.71 ± 0.14	-5.7
	AFG2	0.50	0.60 ± 0.01	20.5
		5.00	4.56 ± 0.29	-8.8
	AFM1	0.50	0.48 ± 0.03	-4.3
		5.00	4.63 ± 0.09	-7.5
	AFM2	5.00	5.04 ± 0.15	0.7
Stability in matrix	AFB1	0.50	0.44 ± 0.01	-12.6
≤- 15 °C		5.00	4.47 ± 0.04	-10.7
9 days	AFB2	0.50	0.46 ± 0.09	-7.6
(n = 3)		5.00	5.63 ± 0.21	12.5
	AFG1	0.50	0.38 ± 0.03	-24.6
		5.00	3.64 ± 0.11	-27.2
	AFG2	0.50	0.40 ± 0.03	-19.2
		5.00	3.93 ± 0.10	-21.4
	AFM1	0.50	0.48 ± 0.01	-3.9
		5.00	4.62 ± 0.13	-7.6

Table S6. Results of the within-run and between-run precision and accuracy evaluation for the analysis of aflatoxins in cattle milk.

Component	Theoretical concentration (ng mL^{-1})	Mean concentration ± SD (ng mL^{-1})	Precision, RSD (%)	Accuracy (%)
AFB1	0.050 [a]	0.048 ± 0.003	6.9	-3.2
	0.050 [b]	0.045 ± 0.005	11.2	-10.0
	0.50 [a]	0.50 ± 0.01	2.2	-0.5
	0.50 [b]	0.49 ± 0.02	4.3	-1.6
	5.00 [a]	4.77 ± 0.15	3.1	-4.6
	5.00 [b]	5.04 ± 0.29	5.8	0.9
AFB2	0.050 [a]	0.048 ± 0.007	14.3	-4.5
	0.050 [b]	0.034 ± 0.012	36.1	-31.5
	0.50 [a]	0.49 ± 0.06	11.4	-1.2
	0.50 [b]	0.42 ± 0.07	16.3	-16.7
	5.00 [a]	4.52 ± 0.37	8.2	-9.5
	5.00 [b]	4.20 ± 0.45	10.7	-16.0
AFG1	0.050 [a]	0.046 ± 0.002	3.7	-7.4
	0.050 [b]	0.051 ± 0.005	9.3	0.9
	0.50 [a]	0.48 ± 0.04	8.5	-4.1
	0.50 [b]	0.48 ± 0.06	12.6	-4.0
	5.00 [a]	4.50 ± 0.30	6.7	-9.9
	5.00 [b]	4.86 ± 0.41	8.5	-2.9
AFG2	0.050 [a]	0.037 ± 0.003	8.6	-26.7
	0.050 [b]	0.042 ± 0.011	27.3	-16.1
	0.50 [a]	0.49 ± 0.04	7.5	-1.3
	0.50 [b]	0.49 ± 0.03	5.8	-1.5
	5.00 [a]	4.77 ± 0.17	3.6	-4.6
	5.00 [b]	4.86 ± 0.36	7.4	-2.7
AFM1	0.025 [a]	0.023 ± 0.003	11.9	-7.3
	0.025 [b]	0.026 ± 0.005	18.9	4.0
	0.050 [a]	0.042 ± 0.006	14.1	-15.3
	0.050 [b]	0.048 ± 0.007	14.6	-4.5
	0.50 [a]	0.50 ± 0.02	4.6	-0.8
	0.50 [b]	0.49 ± 0.02	4.8	-2.0
	5.00 [a]	4.73 ± 0.13	2.8	-5.4
	5.00 [b]	4.87 ± 0.23	4.7	-2.6
AFM2	0.50 [a]	0.49 ± 0.07	14.1	-3.0
	0.50 [b]	0.37 ± 0.11	30.6	-26.2
	5.00 [a]	4.16 ± 0.18	4.4	-16.9
	5.00 [b]	4.43 ± 0.73	16.6	-11.3

Note: [a] Within-run accuracy and precision (n=6); [b] Between-run accuracy and precision (n= 3 x 6); SD: standard deviation; RSD: relative standard deviation; Acceptance criteria: accuracy: < 1 ng mL-1: -50% to +20%, ≥ 1 to < 10 ng mL^{-1}: -40% to +20%, ≥ 10 to < 100 ng mL^{-1}: -30% to +20%; within-run precision (RSD_{max}): < 1 ng mL^{-1}: 30 %, ≥1 to < 10 ng mL^{-1}: 25.0%, ≥ 10 to < 100 ng mL^{-1}: 15%; between-run precision: < 1 ng mL^{-1}: 45%, ≥1 to < 10 ng mL^{-1}: 32%, ≥ 10 to < 100 ng mL^{-1}: 23% [VICH GL49].

Table S7. Results of the stability evaluation of aflatoxins in milk sample extracts (stored at 2 - 8 °C) and in milk during 3 freeze-thaw cycles and during storage at ≤- 15 °C.

Storage conditions	Component	Theoretical concentration (ng mL^{-1})	Mean concentration ± SD (ng mL^{-1})	Accuracy (%)
Stability in extract	AFB1	0.50	0.51 ± 0.02	2.0
2-8 °C		5.00	5.82 ± 0.19	16.3
20 days	AFB2	0.50	0.54 ± 0.02	8.9
		5.00	5.44 ± 0.06	8.7
	AFG1	0.50	0.50 ± 0.01	-0.5
		5.00	5.37 ± 0.05	7.4
(n = 3)	AFG2	0.50	0.60 ± 0.03	20.1
		5.00	6.16 ± 0.14	23.2
	AFM1	0.50	0.54 ± 0.02	7.6
		5.00	5.57 ± 0.11	11.5
	AFM2	0.50	0.53 ± 0.05	5.7
		5.00	4.59 ± 0.12	-8.1
Freeze-thaw stability	AFB1	0.50	0.47 ± 0.03	-5.6
≤- 15 °C to room temperature		5.00	5.11 ± 0.21	2.3
3 cycles	AFB2	0.50	0.49 ± 0.04	-2.3
(n = 3)		5.00	5.10 ± 0.29	2.1
	AFG1	0.50	0.49 ± 0.03	-2.3
		5.00	5.20 ± 0.14	4.0
	AFG2	0.50	0.48 ± 0.01	-4.4
		5.00	4.99 ± 0.22	-0.2
	AFM1	0.50	0.52 ± 0.01	3.5
		5.00	5.27 ± 0.22	5.4
	AFM2	0.50	0.48 ± 0.05	-4.7
		5.00	4.77 ± 0.25	-4.6

Table S8. Results of the within-run and between-run precision and accuracy evaluation for the analysis of aflatoxins in cattle ruminal fluid.

Component	Theoretical concentration (ng mL^{-1})	Mean concentration ± SD (ng mL^{-1})	Precision, RSD (%)	Accuracy (%)
AFB1	0.100 [a]	0.096 ± 0.011	11.1	-3.6
	0.100 [b]	0.095 ± 0.015	15.6	-4.6
	0.50 [a]	0.46 ± 0.02	4.5	-8.0
	0.50 [b]	0.48 ± 0.04	7.6	-3.9
	5.00 [a]	4.85 ± 0.17	3.6	-3.0
	5.00 [b]	4.99 ± 0.29	5.7	-0.1
AFB2	0.100 [a]	0.104 ± 0.021	20.4	4.1
	0.100 [b]	0.093 ± 0.019	20.8	-7.2
	0.50 [a]	0.49 ± 0.05	9.9	-1.9
	0.50 [b]	0.46 ± 0.04	8.6	-7.4
	5.00 [a]	4.45 ± 0.13	2.9	-11.0
	5.00 [b]	5.00 ± 0.35	6.9	0.0
AFG1	0.100 [a]	0.104 ± 0.009	8.8	3.5
	0.100 [b]	0.090 ± 0.021	23.0	-10.1
	0.50 [a]	0.51 ± 0.03	5.0	2.0
	0.50 [b]	0.50 ± 0.03	6.4	-0.7
	5.00 [a]	5.29 ± 0.36	6.7	5.8
	5.00 [b]	5.06 ± 0.35	7.0	1.2
AFG2	0.10 [a]	0.099 ± 0.014	14.2	-0.8
	0.10 [b]	0.092 ± 0.022	24.3	-7.8
	0.50 [a]	0.48 ± 0.04	8.0	-3.9
	0.50 [b]	0.48 ± 0.09	19.0	-4.7
	5.00 [a]	4.57 ± 0.15	3.3	-8.6
	5.00 [b]	4.92 ± 0.41	8.3	-1.6
AFM1	0.100 [a]	0.100 ± 0.008	7.9	0.1
	0.100 [b]	0.106 ± 0.011	10.7	5.5
	0.50 [a]	0.48 ± 0.05	11.0	-4.6
	0.50 [b]	0.50 ± 0.05	10.5	-0.
	5.00 [a]	4.83 ± 0.17	3.4	-3.3
	5.00 [b]	5.02 ± 0.35	7.0	0.5
AFM2	0.50 [a]	0.45 ± 0.06	14.2	-9.4
	0.50 [b]	0.43 ± 0.07	15.1	-13.8
	5.00 [a]	4.98 ± 0.14	2.9	-0.4
	5.00 [b]	5.03 ± 0.60	12.0	0.5

Note: [a] Within-run accuracy and precision (n=6); [b] Between-run accuracy and precision (n= 3 x 6); SD: standard deviation; RSD: relative standard deviation; Acceptance criteria: accuracy: < 1 ng mL-1: -50% to +20%, ≥ 1 to < 10 ng mL^{-1}: -40% to +20%, ≥ 10 to < 100 ng mL^{-1}: -30% to +20%; within-run precision (RSD_{max}): < 1 ng mL^{-1}: 30 %, ≥1 to < 10 ng mL^{-1}: 25.0%, ≥ 10 to < 100 ng mL^{-1}: 15%; between-run precision: < 1 ng mL^{-1}: 45%, ≥1 to < 10 ng mL^{-1}: 32%, ≥ 10 to < 100 ng mL^{-1}: 23% [VICH GL49].

Table S9. Results of the stability evaluation of aflatoxins in ruminal fluid sample extracts (stored at 8 °C) and in ruminal fluid during 3 freeze-thaw cycles.

Storage conditions	Component	Theoretical concentration (ng mL^{-1})	Mean concentration ± SD (ng mL^{-1})	Accuracy (%)
Stability in extract	AFB1	0.50	0.55 ± 0.06	10.0
		5.00	5.47 ± 1.01	9.5
2-8 °C	AFB2	0.50	2.48 ± 0.13	-0.7
		5.00	No data available	/
10 days	AFG1	0.50	0.56 ± 0.03	11.6
		5.00	5.49 ± 0.21	9.7
(n = 5)	AFG2	0.50	0.53 ± 0.10	5.3
		5.00	5.40 ± 0.36	8.1
	AFM1	0.50	0.47 ± 0.04	-5.4
		5.00	4.63 ± 0.20	-7.4
	AFM2	0.50	0.58 ± 0.07	15.1
		5.00	5.52 ± 0.06	10.5
Freeze-thaw stability	AFB1	0.50	0.52 ± 0.05	4.6
		5.00	4.95 ± 0.39	-0.9
≤- 15 °C to room temperature	AFB2	0.50	0.52 ± 0.03	5.8
		5.00	4.97 ± 0.31	6.3
3 cycles	AFG1	0.50	0.49 ± 0.04	-1.9
		5.00	4.67 ± 0.24	-6.5
(n = 3)	AFG2	0.50	0.47 ± 0.08	-5.5
		5.00	4.48 ± 0.37	-10.4
	AFM1	0.50	0.36 ± 0.04	-27.3
		5.00	3.05 ± 0.14	38.9
	AFM2	0.50	0.40 ± 0.02	-20.2
		5.00	4.17 ± 0.08	-16.6

Table S10. Results of the evaluation of extraction recovery and matrix effect for the LC-MS/MS analysis of aflatoxins in cattle plasma, milk and ruminal fluid.

Matrix	Component	Spiked concentration (ng mL^{-1})	Extraction recovery (%)	Matrix effect (%)
Plasma	AFB1	0.50	57.5	96.9
		5.00	72.1	83.6
	AFB2	0.50	61.6	91.8
		5.00	76.1	80.1
	AFG1	0.50	59.2	104.9
		5.00	73.3	82.3
	AFG2	0.50	65.7	84.9
		5.00	76.7	74.6
	AFM1	0.50	67.2	98.7
		5.00	80.0	84.2
	AFM2	0.50	63.1	95.2
		5.00	73.6	78.6
Milk	AFB1	0.50	31.3	53.3
		5.00	42.6	59.7
	AFB2	0.50	35.4	64.5
		5.00	47.8	68.5
	AFG1	0.50	33.8	56.0
		5.00	42.9	62.0
	AFG2	0.50	35.2	66.5
		5.00	47.0	70.9
	AFM1	0.50	34.9	74.7
		5.00	46.6	80.8
	AFM2	0.50	24.7	67.4
		5.00	36.3	77.5
Ruminal fluid	AFB1	0.50	70.9	21.8
		5.00	69.1	22.8
	AFB2	0.50	64.0	36.7
		5.00	65.9	35.7
	AFG1	0.50	67.3	28.6
		5.00	69.8	24.8
	AFG2	0.50	75.1	37.2
		5.00	68.5	31.7
	AFM1	0.50	64.1	33.2
		5.00	68.9	34.7
	AFM2	0.50	61.4	24.3
		5.00	65.7	29.5

Table S11. Results of the within-run and between-run precision and accuracy evaluation for the analysis of aflatoxins in chicken liver.

Component	Theoretical concentration (μg kg^{-1})	Mean concentration ± SD (μg kg^{-1})	Precision, RSD (%)	Accuracy (%)
AFB1	0.050 [a]	0.044 ± 0.003	7.3	-12.4
	0.050 [b]	0.045 ± 0.005	10.6	-9.6
	0.50 [a]	0.49 ± 0.06	11.6	-2.9
	0.50 [b]	0.50 ± 0.09	18.2	-0.9
	5.00 [a]	4.95 ± 0.24	4.9	1.0
	5.00 [b]	5.07 ± 0.47	9.3	1.4
AFB2	0.10 [a]	0.09 ± 0.01	14.8	-8.6
	0.10 [b]	0.11 ± 0.03	26.7	7.7
	0.50 [a]	0.51 ± 0.07	14.4	2.1
	0.50 [b]	0.49 ± 0.05	9.7	-2.2
	5.00 [a]	4.89 ± 0.40	8.1	-2.1
	5.00 [b]	5.00 ± 0.60	12.1	0.0
AFG1	0.25 [a]	0.25 ± 0.07	28.5	0.1
	0.25 [b]	0.23 ± 0.05	22.0	-9.0
	0.50 [a]	0.50 ± 0.07	13.1	0.2
	0.50 [b]	0.48 ± 0.07	13.8	-4.0
	5.00 [a]	5.16 ± 0.65	12.7	3.2
	5.00 [b]	5.16 ± 0.59	11.5	3.3
AFG2	0.25 [a]	0.24 ± 0.04	17.4	-2.4
	0.25 [b]	0.20 ± 0.05	23.6	-18.1
	0.50 [a]	0.44 ± 0.06	12.6	-12.9
	0.50 [b]	0.48 ± 0.07	15.0	-4.8
	5.00 [a]	4.28 ± 0.30	6.9	-14.4
	5.00 [b]	4.87 ± 0.86	17.7	-2.5
AFM1	0.010 [a]	0.102 ± 0.004	4.2	1.6
	0.010 [b]	0.099 ± 0.009	9.2	-0.7
	0.50 [a]	0.51 ± 0.10	18.9	1.8
	0.50 [b]	0.51 ± 0.06	12.6	1.0
	5.00 [a]	4.86 ± 0.09	1.9	-2.8
	5.00 [b]	4.94 ± 0.50	10.1	-1.2
AFM2	0.50 [a]	0.48 ± 0.03	6.2	-4.6
	0.50 [b]	0.46 ± 0.04	9.1	-8.2
	5.00 [a]	4.51 ± 0.32	7.2	-9.7
	5.00 [b]	4.65 ± 0.48	10.2	-7.0

Note: [a] Within-run accuracy and precision (n=6); [b] Between-run accuracy and precision (n= 3 x 6); SD: standard deviation; RSD: relative standard deviation; Acceptance criteria: accuracy: < 1 μg kg^{-1}: -50% to +20%, ≥ 1 to < 10 μg kg^{-1}: -40% to +20%, ≥ 10 to < 100 μg kg^{-1}: -30% to +20%; within-run precision (RSD_{max}): < 1 μg kg^{-1}: 30 %, ≥1 to < 10 μg kg^{-1}: 25.0%, ≥ 10 to < 100 μg kg^{-1}: 15%; between-run precision: < 1 μg kg^{-1}: 45%, ≥1 to < 10 μg kg^{-1}: 32%, ≥ 10 to < 100 μg kg^{-1}: 23% [VICH GL49].

Table S12. Results of the stability evaluation of aflatoxins in chicken liver sample extracts (stored at 8 °C), in chicken liver during 3 freeze-thaw cycles and during storage at ≤- 15 °C.

Storage conditions	Component	Theoretical concentration (μg kg^{-1})	Mean concentration ± SD (μg kg^{-1})	Accuracy (%)
Stability in extract<br>8 °C<br>36 days<br>(n = 3)	AFB1	0.50	0.43 ± 0.16	-14.1
		5.00	5.28 ± 0.90	5.6
	AFB2	0.50	0.60 ± 0.06	19.2
		5.00	5.61 ± 1.05	12.2
	AFG1	0.50	0.15 ± 0.08	-70.7
		5.00	1.80 ± 0.53	-64.0
	AFG2	0.50	0.78 ± 0.11	55.4
		5.00	8.13 ± 1.91	62.6
	AFM1	0.50	0.60 ± 0.02	20.2
		5.00	6.65 ± 0.11	32.9
	AFM2	5.00	3.58 ± 0.14	-28.5
Freeze-thaw stability<br>≤- 15 °C to room temperature<br>3 cycles<br>(n = 3)	AFB1	0.50	0.44 ± 0.05	-12.7
		5.00	4.50 ± 0.24	-10.1
	AFB2	0.50	0.43 ± 0.09	-13.6
		5.00	4.65 ± 0.49	-7.0
	AFG1	0.50	0.52 ± 0.04	3.9
		5.00	5.21 ± 0.27	4.2
	AFG2	0.50	0.52 ± 0.02	4.4
		5.00	4.91 ± 0.24	-1.7
	AFM1	0.50	0.42 ± 0.03	-15.3
		5.00	4.28 ± 0.13	-14.5
	AFM2	5.00	4.23 ± 0.0.11	-15.5

Table S13. Results of the within-run and between-run precision and accuracy evaluation for the analysis of aflatoxins in chicken muscle.

Component	Theoretical concentration (μg kg^{-1})	Mean concentration ± SD (μg kg^{-1})	Precision, RSD (%)	Accuracy (%)
AFB1	0.050 [a]	0.049 ± 0.007	15.3	-2.5
	0.050 [b]	0.038 ± 0.012	32.0	-23.0
	0.50 [a]	0.52 ± 0.03	5.0	4.0
	0.50 [b]	0.49 ± 0.05	9.2	-1.1
	5.00 [a]	5.19 ± 0.31	5.9	3.8
	5.00 [b]	5.18 ± 0.70	13.5	3.6
AFB2	0.25 [a]	0.24 ± 0.03	10.7	-5.5
	0.24 [b]	0.24 ± 0.04	16.4	-2.6
	0.50 [a]	0.49 ± 0.04	8.9	-1.3
	0.50 [b]	0.49 ± 0.04	8.0	-1.8
	5.00 [a]	4.99 ± 0.28	5.6	-0.2
	5.00 [b]	4.84 ± 0.41	8.5	-3.3
AFG1	0.25 [a]	0.26 ± 0.01	4.9	3.7
	0.25 [b]	0.24 ± 0.03	11.6	-5.2
	0.50 [a]	0.51 ± 0.03	5.0	1.5
	0.50 [b]	0.48 ± 0.04	8.7	-3.4
	5.00 [a]	5.11 ± 0.20	4.0	2.2
	5.00 [b]	5.03 ± 0.39	7.8	0.5
AFG2	0.25 [a]	0.25 ± 0.03	13.2	1.0
	0.25 [b]	0.26 ± 0.06	22.0	3.9
	0.50 [a]	0.48 ± 0.06	12.2	-3.8
	0.50 [b]	0.51 ± 0.05	10.4	2.4
	5.00 [a]	5.12 ± 0.22	4.3	2.3
	5.00 [b]	5.16 ± 0.47	9.1	3.2
AFM1	0.10 [a]	0.08 ± 0.01	9.1	-16.1
	0.10 [b]	0.09 ± 0.01	9.9	-8.3
	0.50 [a]	0.51 ± 0.03	6.2	1.2
	0.50 [b]	0.50 ± 0.04	8.4	0.40
	5.00 [a]	5.11 ± 0.33	6.4	2.3
	5.00 [b]	5.00 ± 0.51	10.1	0.1

Note: [a] Within-run accuracy and precision (n=6); [b] Between-run accuracy and precision (n= 3 x 6); SD: standard deviation; RSD: relative standard deviation; Acceptance criteria: accuracy: < 1 μg kg^{-1}: -50% to +20%, ≥ 1 to < 10 μg kg^{-1}: -40% to +20%, ≥ 10 to < 100 μg kg^{-1}: -30% to +20%; within-run precision (RSD_{max}): < 1 μg kg^{-1}: 30 %, ≥1 to < 10 μg kg^{-1}: 25.0%, ≥ 10 to < 100 μg kg^{-1}: 15%; between-run precision: < 1 μg kg^{-1}: 45%, ≥1 to < 10 μg kg^{-1}: 32%, ≥ 10 to < 100 μg kg^{-1}: 23% [VICH GL49].

Table S14. Results of the stability evaluation of aflatoxins in chicken muscle sample extracts (stored at 8 °C), in chicken muscle during 3 freeze-thaw cycles and during storage at ≤- 15 °C.

Storage conditions	Component	Theoretical concentration (μg kg^{-1})	Mean concentration ± SD (μg kg^{-1})	Accuracy (%)
Stability in extract	AFB1	0.50	0.40 ± 0.1	-20.8
2 - 8 °C		5.00	4.24 ± 0.11	-15.3
28 days	AFB2	0.50	0.41 ± 0.01	-18.4
(n = 3)		5.00	5.00 ± 0.68	0.1
	AFG1	0.50	0.41 ± 0.03	-18.1
		5.00	5.37 ± 0.05	7.4
	AFG2	0.50	0.48 ± 0.00	-4.2
		5.00	6.66 ± 0.76	33.3
	AFM1	0.50	0.51 ± 0.05	2.7
		5.00	4.88 ± 0.12	-2.3
Freeze-thaw stability	AFB1	0.50	0.44 ± 0.02	-12.4
≤- 15 °C to room temperature		5.00	5.12 ± 0.36	2.4
3 cycles	AFB2	0.50	0.43 ± 0.03	-14.8
(n = 3)		5.00	4.93 ± 0.19	-1.4
	AFG1	0.50	0.44 ± 0.02	-12.0
		5.00	4.70 ± 0.06	-6.1
	AFG2	0.50	0.50 ± 0.01	-0.3
		5.00	4.60 ± 0.44	-8.0
	AFM1	0.50	0.48 ± 0.03	-3.0
		5.00	4.69 ± 0.42	-6.2

Table S15. Results of the within-run and between-run precision and accuracy evaluation for the analysis of aflatoxins in chicken eggs.

Component	Theoretical concentration (μg kg^{-1})	Mean concentration ± SD (μg kg^{-1})	Precision, RSD (%)	Accuracy (%)
AFB1	0.025[a]	0.027 ± 0.002	7.3	6.7
	0.025[b]	0.025 ± 0.003	11.9	-0.7
	0.050 [a]	0.046 ± 0.007	15.7	-8.4
	0.050 [b]	0.052 ± 0.011	21.1	4.6
	0.50 [a]	0.48 ± 0.01	2.8	-4.9
	0.50 [b]	0.47 ± 0.05	9.6	-5.4
	5.00 [a]	5.29 ± 0.18	3.4	5.8
	5.00 [b]	4.59 ± 1.25	27.2	-8.3
AFB2	0.025[a]	0.027 ± 0.001	4.0	7.6
	0.025[b]	0.026 ± 0.003	10.2	2.7
	0.050 [a]	0.048 ± 0.012	25.1	-4.4
	0.050 [b]	0.053 ± 0.012	23.2	5.2
	0.50 [a]	0.49 ± 0.02	4.8	-2.3
	0.50 [b]	0.49 ± 0.04	7.1	-1.1
	5.00 [a]	5.32 ± 0.15	2.8	6.4
	5.00 [b]	5.24 ± 0.91	17.3	4.9
AFG1	0.050 [a]	0.050 ± 0.010	13.0	4.7
	0.050 [b]	0.048 ± 0.011	22.9	-4.3
	0.50 [a]	0.44 ± 0.03	6.0	-11.6
	0.50 [b]	0.45 ± 0.04	9.7	-9.6
	5.00 [a]	4.94 ± 0.23	4.7	-1.1
	5.00 [b]	4.47 ± 0.69	15.4	-10.6
AFG2	0.050 [a]	0.058 ± 0.008	13.4	15.3
	0.050 [b]	0.056 ± 0.013	22.7	12.2
	0.50 [a]	0.52 ± 0.02	3.4	4.0
	0.50 [b]	0.53 ± 0.04	7.9	5.3
	5.00 [a]	4.98 ± 0.54	10.9	-0.3
	5.00 [b]	5.37 ± 0.56	10.4	7.5
AFM1	0.025[a]	0.029 ± 0.001	4.9	14.7
	0.025[b]	0.026 ± 0.003	11.9	2.4
	0.050 [a]	0.057 ± 0.011	19.5	13.7
	0.050 [b]	0.053 ± 0.010	19.1	6.8
	0.50 [a]	0.51 ± 0.04	7.8	2.9
	0.50 [b]	0.52 ± 0.05	8.9	4.9
	5.00 [a]	5.06 ± 0.56	11.0	1.2
	5.00 [b]	5.34 ± 0.90	16.8	6.9
AFM2	0.50 [a]	0.57 ± 0.04	6.9	14.6
	0.50 [b]	0.54 ± 0.06	10.6	9.1
	5.00 [a]	4.76 ± 0.16	3.4	-4.7
	5.00 [b]	5.28 ± 0.83	15.7	5.6

Note: [a] Within-run accuracy and precision (n=6); [b] Between-run accuracy and precision (n= 3 x 6); SD: standard deviation; RSD: relative standard deviation; Acceptance criteria: accuracy: < 1 μg kg^{-1}: -50% to +20%, ≥ 1 to < 10 μg kg^{-1}: -40% to +20%, ≥ 10 to < 100 μg kg^{-1}: -30% to +20%; within-run precision (RSD_{max}): < 1 μg kg^{-1}: 30 %, ≥1 to < 10 μg kg^{-1}: 25.0%, ≥ 10 to < 100 μg kg^{-1}: 15%; between-run precision: < 1 μg kg^{-1}: 45%, ≥1 to < 10 μg kg^{-1}: 32%, ≥ 10 to < 100 μg kg^{-1}: 23% [VICH GL49].

Table S16. Results of the stability evaluation of aflatoxins in chicken egg sample extracts (stored at 8 °C), in chicken eggs during 3 freeze-thaw cycles and during storage at ≤- 15 °C.

Storage conditions	Component	Theoretical concentration (μg kg^{-1})	Mean concentration ± SD (μg kg^{-1})	Accuracy (%)
Stability in extract 2 - 8 °C 7 days (n = 3)	AFB1	0.050	0.055 ± 0.002	10.2
		0.50	0.52 ± 0.01	2.1
	AFB2	0.050	0.056 ± 0.002	12.4
		0.50	0.52 ± 0.02	4.5
	AFG1	0.050	0.062 ± 0.004	24.9
		0.50	0.53 ± 0.02	5.8
	AFG2	0.050	0.058 ± 0.003	16.6
		0.50	0.52 ± 0.00	3.6
	AFM1	0.050	0.058 ± 0.006	16.2
		0.50	0.52 ± 0.04	3.7
	AFM2	0.50	0.52 ± 0.18	4.7
Freeze-thaw stability ≤- 15 °C to room temperature 3 cycles (n = 3)	AFB1	0.50	0.34 ± 0.05	-32.3
		5.00	3.81 ± 0.40	-23.8
	AFB2	0.50	0.48 ± 0.01	-4.5
		5.00	4.84 ± 0.19	-3.2
	AFG1	0.50	0.37 ± 0.03	-26.3
		5.00	4.07 ± 0.26	-18.5
	AFG2	0.50	0.44 ± 0.03	-12.9
		5.00	4.70 ± 0.07	-6.1
	AFM1	0.50	0.40 ± 0.03	-20.7
		5.00	4.27 ± 0.14	-14.5

Table S17. Results of the evaluation of extraction recovery and matrix effect for the LC-MS/MS analysis of aflatoxins in chicken plasma, liver, muscle and eggs.

Matrix	Component	Spiked concentration (ng mL^{-1}/ µg kg^{-1})	Extraction recovery (%)	Matrix effect (%)
Plasma	AFB1	0.50	69.5	60.3
		5.00	68.5	66.8
	AFB2	0.50	69.2	69.3
		5.00	71.8	60.2
	AFG1	0.50	70.7	60.4
		5.00	71.7	61.1
	AFG2	0.50	66.1	61.5
		5.00	72.1	68.0
	AFM1	0.50	68.7	79.6
		5.00	70.7	88.5
	AFM2	0.50	71.4	70.6
		5.00	73.5	67.9
liver	AFB1	0.50	32.9	28.0
		5.00	29.7	35.3
	AFB2	0.50	39.3	45.8
		5.00	32.2	55.3
	AFG1	0.50	36.0	42.6
		5.00	33.8	44.9
	AFG2	0.50	38.0	54.6
		5.00	36.2	59.8
	AFM1	0.50	34.6	68.4
		5.00	38.6	79.1
	AFM2	0.50	28.5	66.4
		5.00	32.5	77.4
Muscle	AFB1	0.50	127.3	61.7
		5.00	135.8	61.4
	AFB2	0.50	127.6	79.1
		5.00	142.5	73.4
	AFG1	0.50	114.0	82.0
		5.00	136.2	75.2
	AFG2	0.50	123.0	88.5
		5.00	138.6	78.0
	AFM1	0.50	121.7	74.0
		5.00	135.0	68.1
	AFM2	0.50	16.9	89.0
		5.00	16.3	78.8
Eggs	AFB1	0.50	20.7	96.8
		5.00	11.6	119.3
	AFB2	0.50	19.7	101.7
		5.00	10.0	126.2
	AFG1	0.50	23.9	98.6
		5.00	11.2	117.1
	AFG2	0.50	21.2	105.3
		5.00	10.1	126.6
	AFM1	0.50	14.7	115.3
		5.00	8.3	139.5
	AFM2	5.00	7.5	141.8

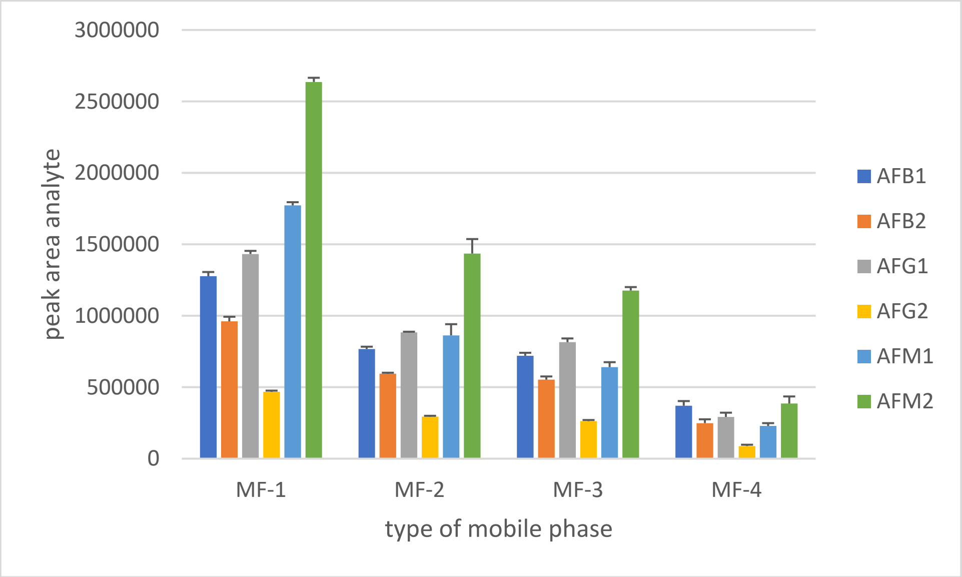

Figure S1. Evaluation of signal intensity (based on peak area) of AFB1, AFB2, AFG1, AFG2, AFM1 and AFM2 during chromatography with different aqueous mobile phases (MF), i.e MF-1 : water; MF-2 : 5 mM NH4FA + 0.1 % FA in water; MF-3 : 10 mM NH4FA + 0.3 % FA in water; MF-4 : 5 mM NH4AA + 0.1 % AA in water. Methanol was used as organic mobile phase in all experiments.
